# Supplementary material for: Benchtop mesoSPIM: a next-generation open-source light-sheet microscope for cleared samples
Source: Nat Commun. 2024 Mar 27;15:2679. doi: 10.1038/s41467-024-46770-2 (PMC10973490; doi:10.1038/s41467-024-46770-2)
Supplement: Supplementary file 1 — Supplementary Information [file 41467_2024_46770_MOESM1_ESM.pdf]

Supplementary Information

Supplementary Figures

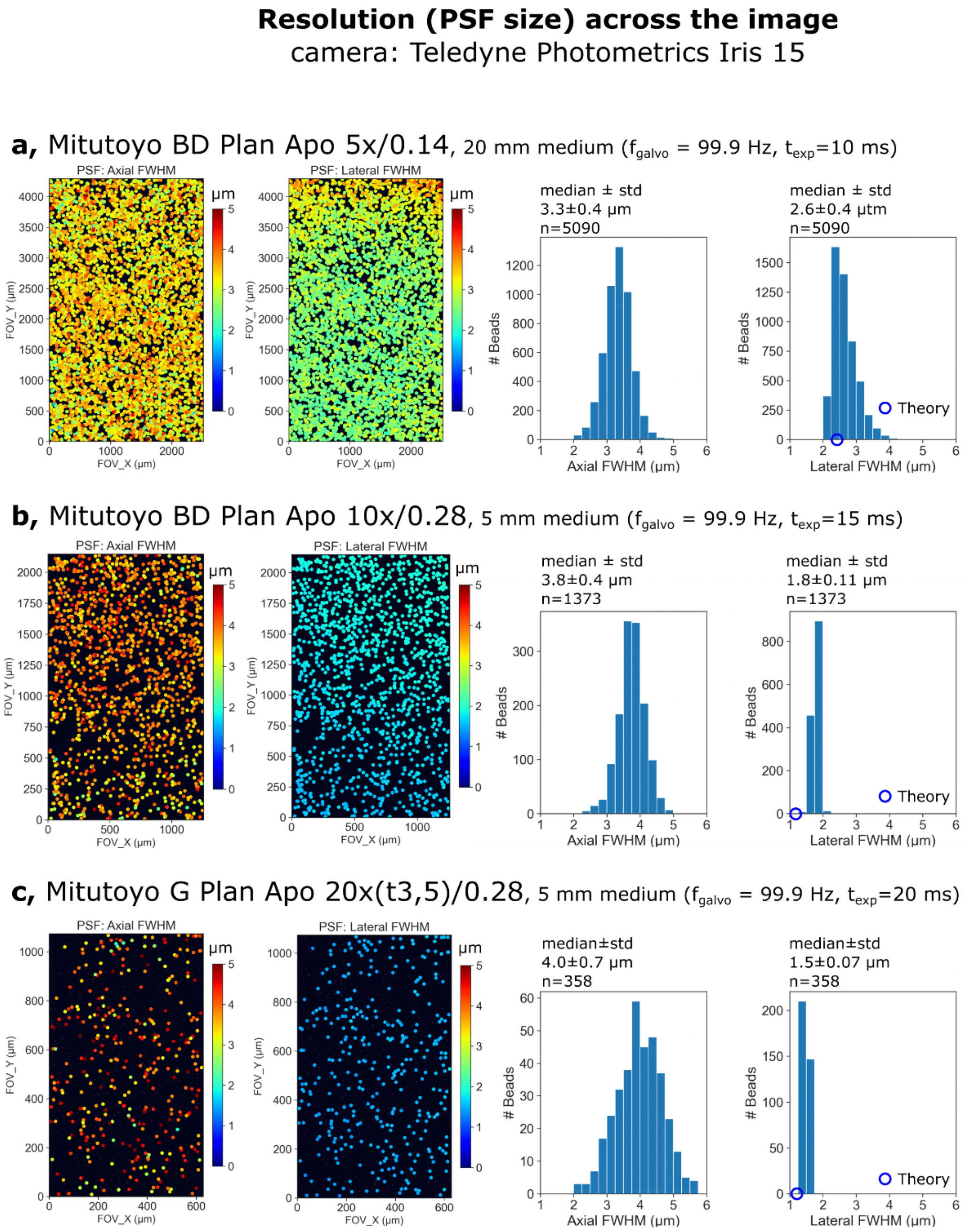

Supplementary Figure 1. **Light-sheet PSF analysis across the field for Mitutoyo Plan Apo detection objectives.** Objectives tested: **a,** Mitutoyo BD Plan Apo 5x/0.14, **b,** Mitutoyo BD Plan Apo 10x/0.28; **c,** Mitutoyo G Plan Apo 20x(t3,5)/0.28. PSF was computed from image stack of dense bead sample (200 nm fluorescent beads) embedded in agarose and index-matched with RI=1.52 medium (CUBIC-R+). The overall thickness of high-index medium (glass, oil, and CUBIC-R+, 5-20 mm, RI=1.52) designated as 'oil' is indicated. Theoretical values for lateral resolution were calculated as $r_{lateral} = 0.61\lambda_{det}/NA_{det}$ (assuming ideal imaging conditions and no refractive index mismatch). Source data for the histograms is provided as Source Data files, raw data of bead images is available at the BioImage Archive (https://www.ebi.ac.uk/biostudies/bioimages/studies/S-BIAD963).

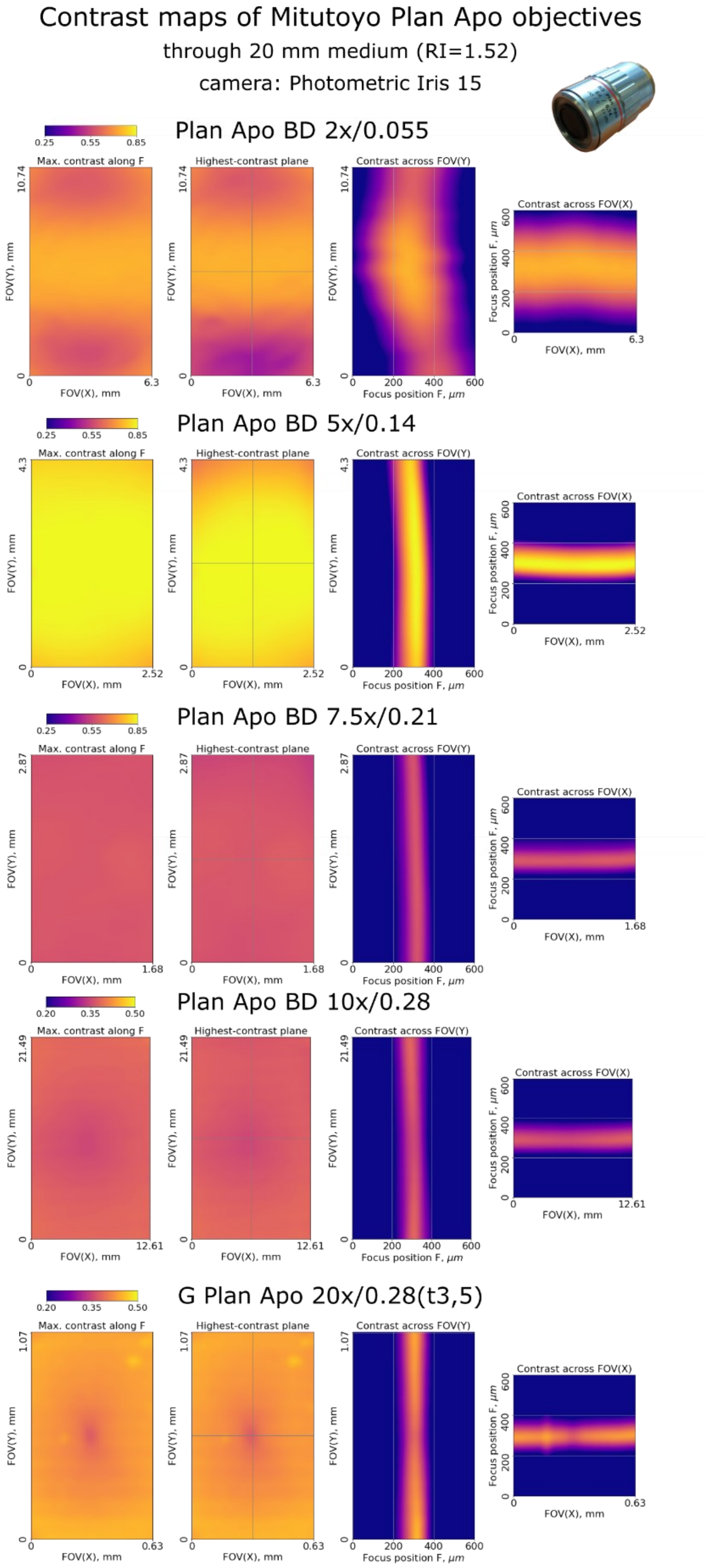

Supplementary Figure 2. **Contrast maps of Mitutoyo Plan Apo objectives.** Imaging of a Ronchi ruling was through 20 mm of high-RI medium (RI 1.52). Note that colorbar scale is adjusted to smaller range for 10x and 20x objectives (NA 0.28) to account for spherical aberration effect due to higher NA. The standard tube lens (Mitutoyo MT-1) was used for tests.

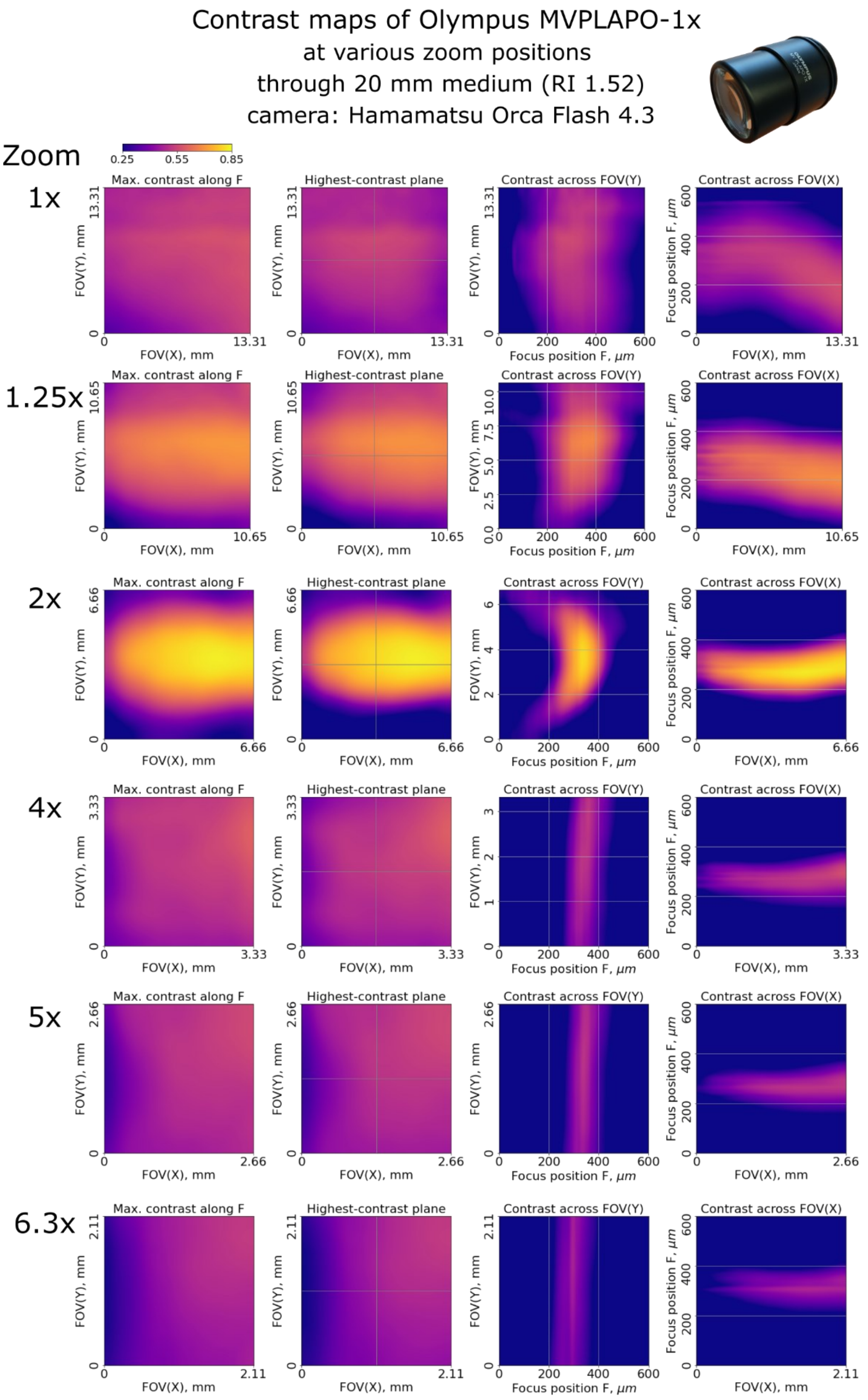

Supplementary Figure 3. **Contrast maps of Olympus MVPLAPO-1x detection objective at various zoom settings.** The zoom was set using the Olympus MVX-10 microscope body (mesoSPIM v.5 configuration). Images of the Ronchi ruling were taken through 20 mm of immersion medium (RI=1.52) to mimic the typical imaging conditions.

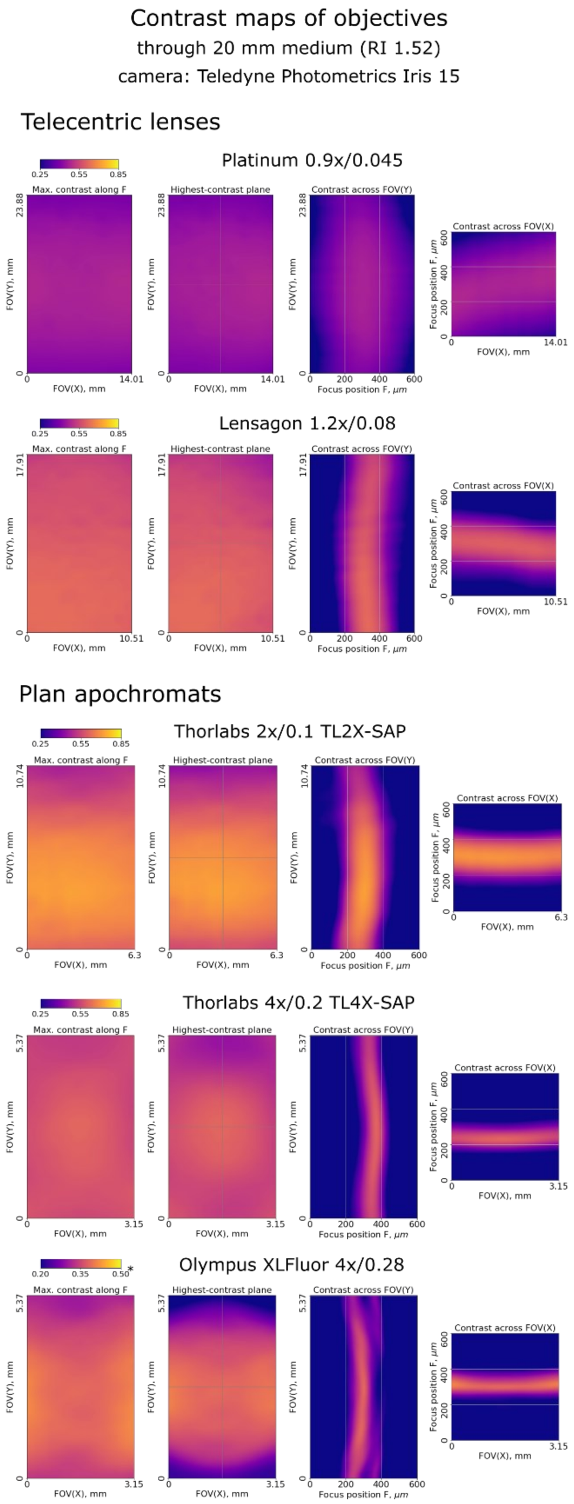

Supplementary Figure 4. **Contrast maps of some detection objectives.** Telecentric lenses: Platinum 0.9x/0.045, Lensagon 1.2x/0.08. Life sciences microscopy objectives: Thorlabs 2x/0.1 TL2x-SAP, Thorlabs 4x/0.2 TL4X-SAP, Olymous XLFluor 4x/0.28. Imaging of Ronchi grating was performed through 20 mm of high-index medium (RI=1.52). Note that colorbar scale is adjusted to smaller range for the Olympus 4x/0.28 to account for spherical aberration effect at higher NA.

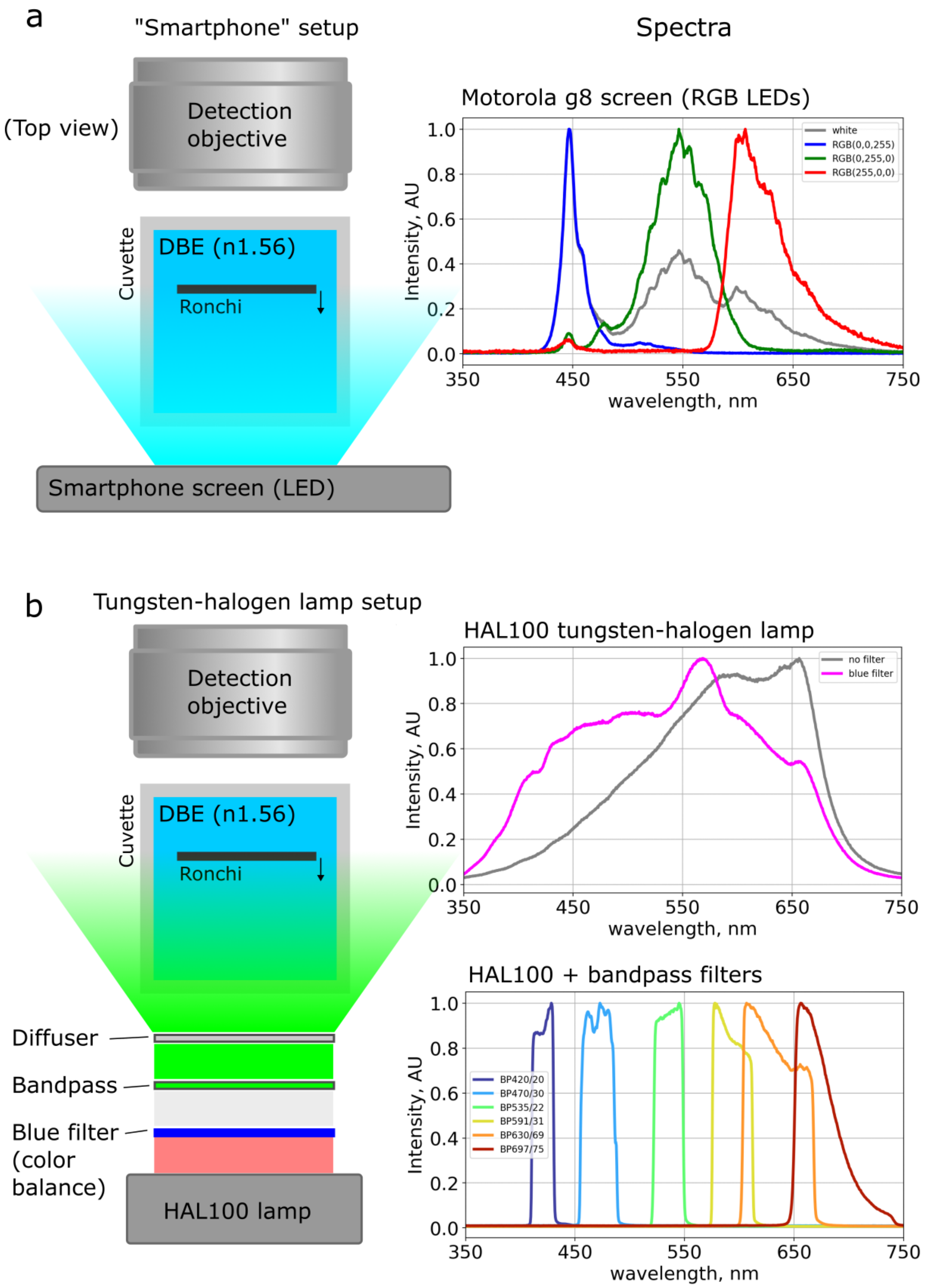

Supplementary Figure 5. Setups and spectra of the light sources used for contrast map testing. a, The basic illumination setup for objective contrast mapping. Left, top view of the testing setup, with the Ronchi slide immersed in a cuvette with DBE and illuminated by the smartphone screen from behind. Right, – spectra of the smartphone screen with uniform RGB slide (white – (255,255,255), etc). **b,** The extended illumination setup used for quantification of the chromatic effects, with a broad-spectrum HAL100 tungsten-halogen lamp and bandbass filters. Left: top view of the testing setup. Right: Spectrum of the tungsten-halogen lamp without and with a blue filter for color balance (top) and spectral bands provided by using the 6 bandpass filters (bottom). Source data for the graphs is provided as Source Data files.

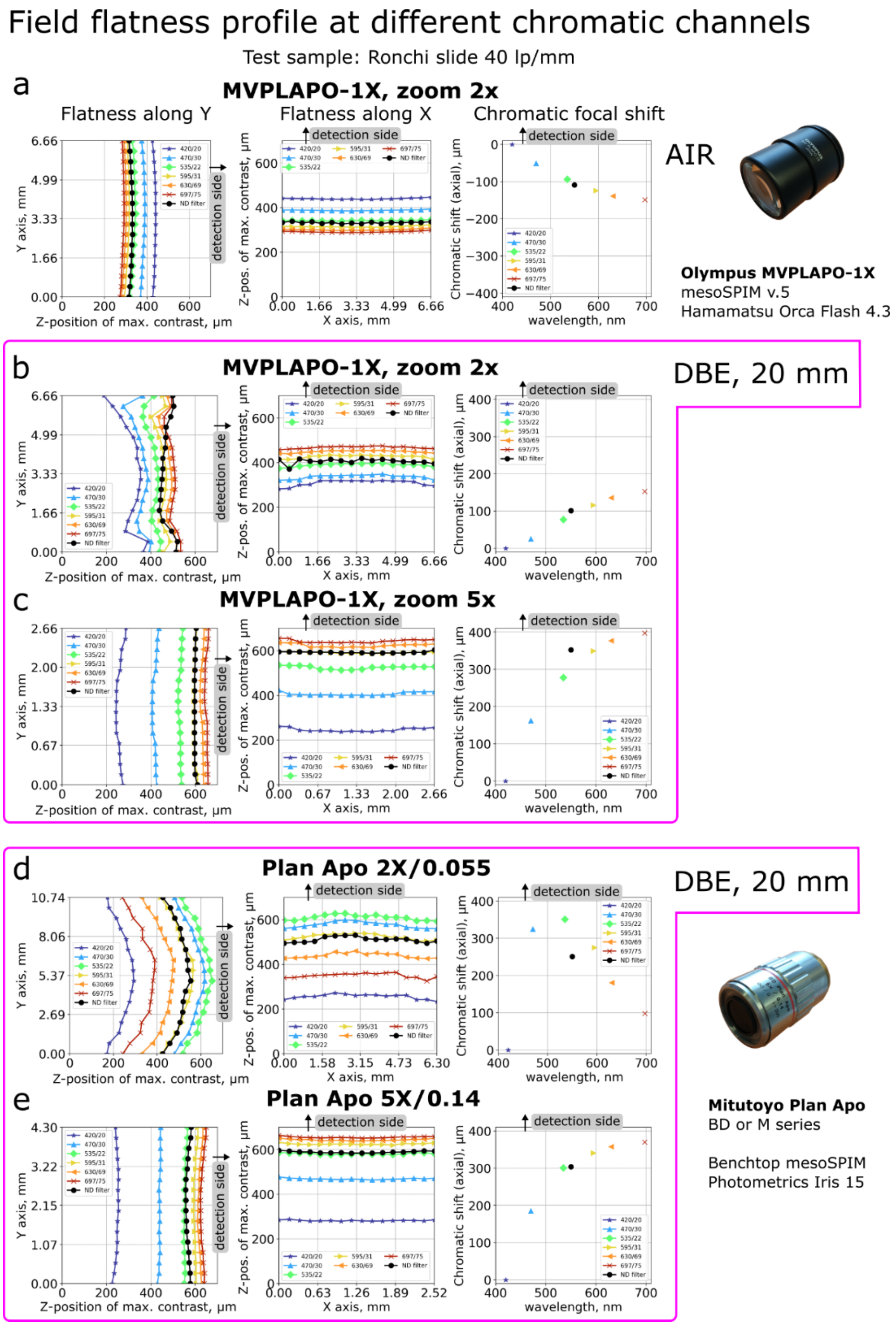

Supplementary Figure 6. Sections of the best-focus surface at different chromatic channels for select plan apochromat objectives. The test target, Ronchi slide 40 lp/mm, was in the air or immersed in DBE medium (20 mm total, including cuvette glass wall). **a,** Olympus MVPLAPO-1x on MVX-10 body at zoom settings 2x and Ronchi slide in the air; **b**, Ronchi slide immersed in DBE; **c,** zoom setting 5x, Ronchi slide in DBE. **d,** Mitutoyo Plan Apo BD 2x/0.055, Ronchi slide in DBE; **e,** Mitutoyo Plan Apo BD 5x/0.14, Ronchi slide in DBE. Note that Mitutoyo objectives were tested with a Photometrics Iris 15 camera, which has sensor significantly larger in Y axis and slightly smaller in X axis than Hamamatsu Orca Flash 4.3 (Figure 2 of the main text). Measurements for Mitutoyo BD also applies to equivalent objectives in M series. In each row, left and middle panels have z-position zero chosen arbitrary, to cover the full range of profiles in all channels; the right panel shows profile offsets relative to the “420/20 nm filter” measurement. Source data for the graphs is provided as Source Data files.

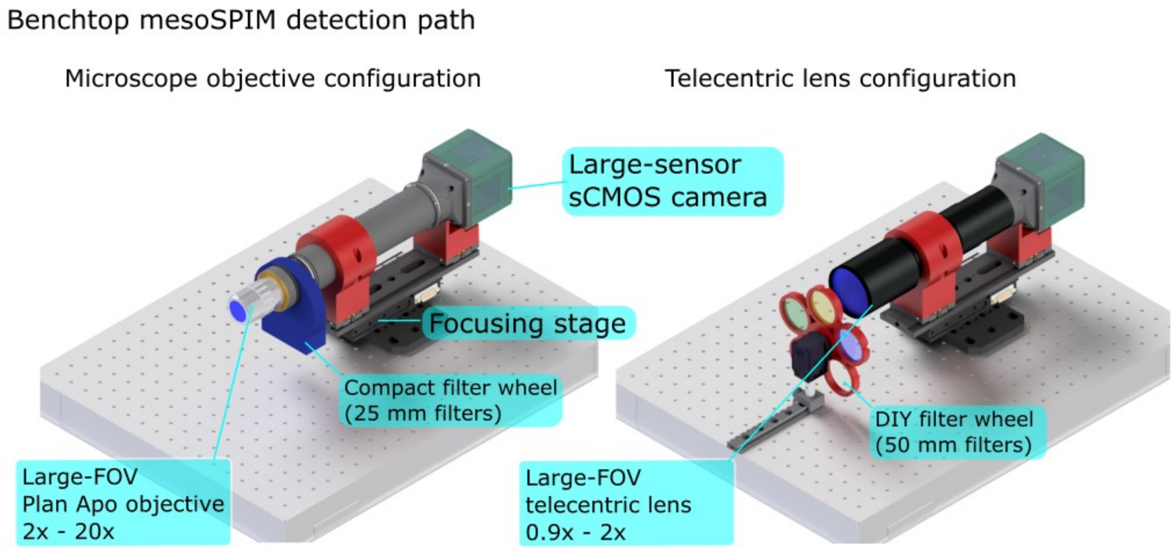

Supplementary Figure 7. The Benchtop mesoSPIM detection path design. CAD drawing of detection arm mechanical design. Two main options are shown: microscope objective (2x – 20x) vs telecentric lens (0.5 – 2x). Custom-made (3D-printed) parts are red.

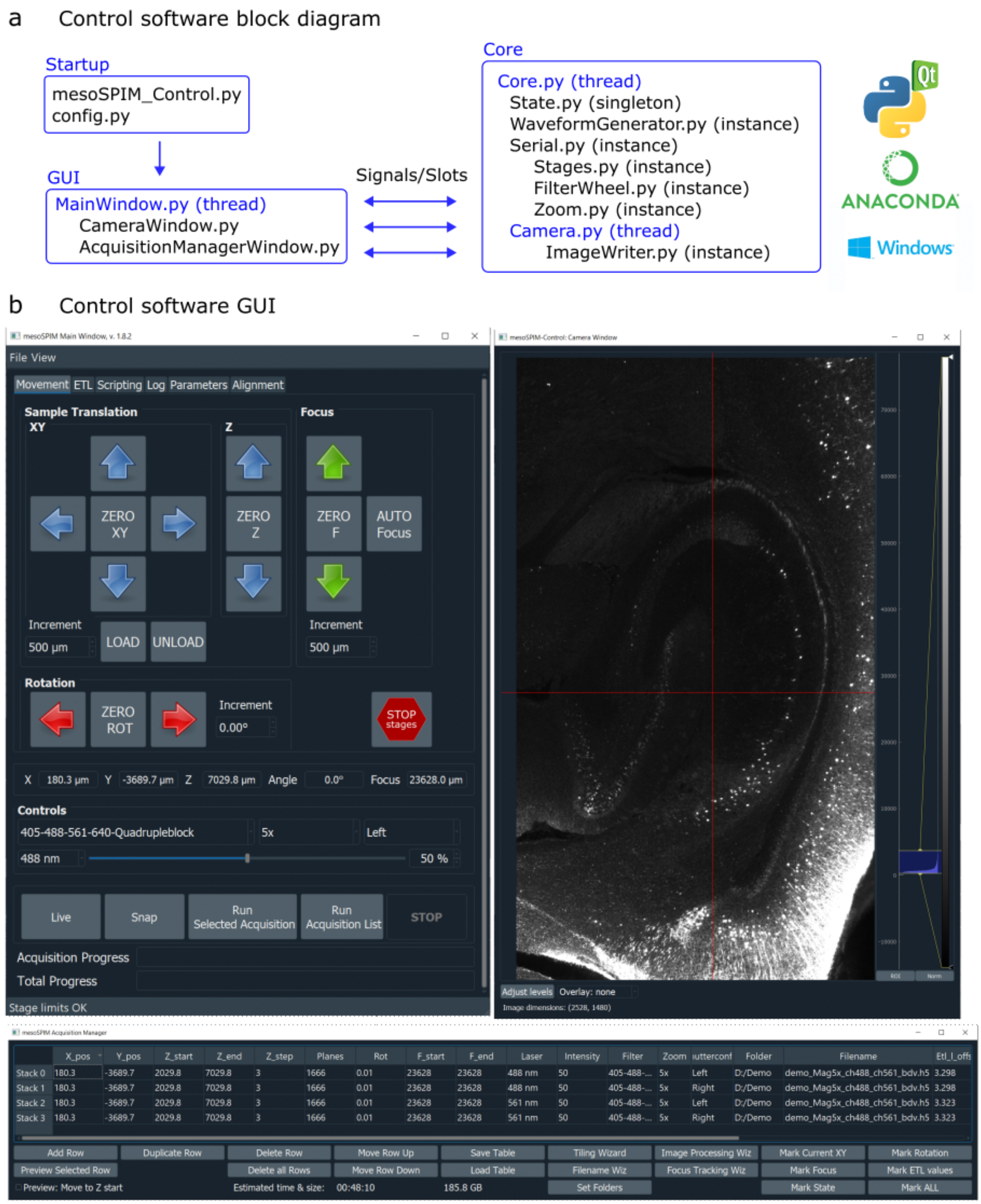

Supplementary Figure 8. The mesoSPIM control software. a, Block diagram: startup file mesoSPIM_Control.py launches system-specific parameters from the config file, creates GUI windows, and initializes core classes and threads. Communication between threads and class instances is managed by PyQt signal/slot mechanisms. Python interpreter and dependencies are managed by Anaconda under Windows 10, 64-bit. **b,** The three main GUI windows, in dark scheme: Main Window with the control parameters, Camera Window with live view of the current imaging plane, and Acquisition Manager which contains all required parameters for each stack/view acquisition.

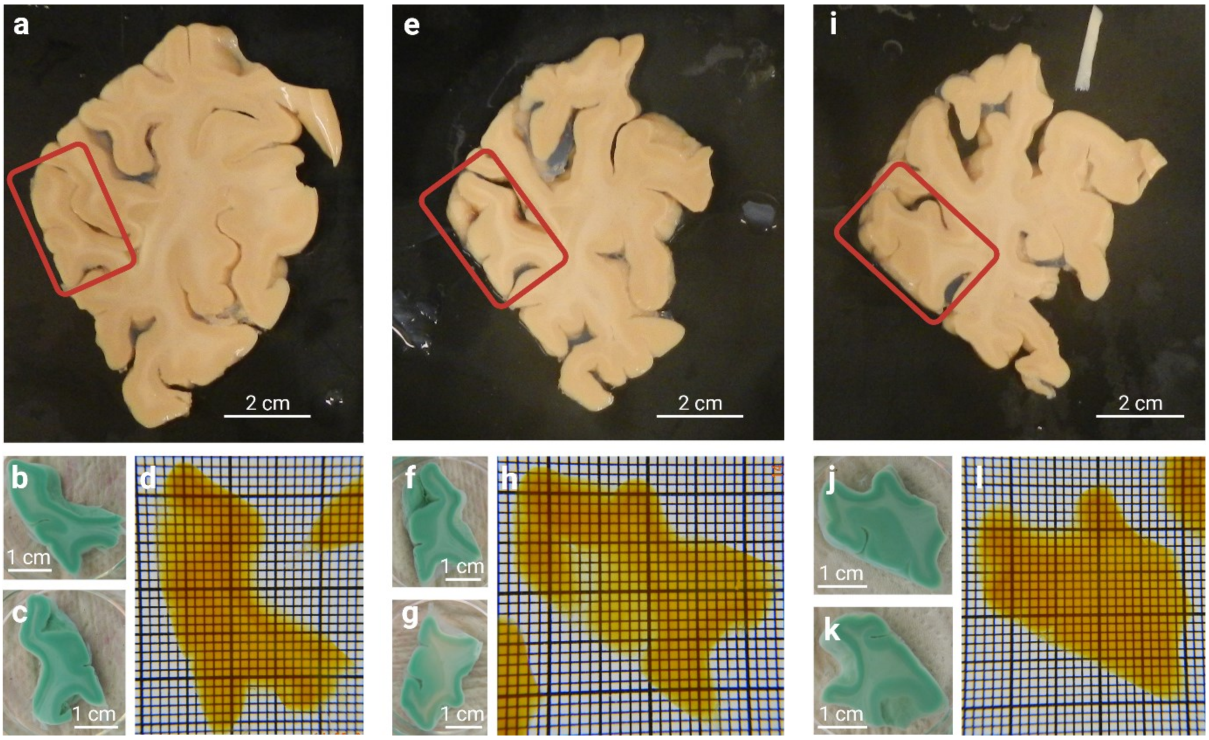

Supplementary Figure 9: Histological processing of Occipital lobe 1 samples with MASH-MB. Whole occipital lobe slices shown with the anterior side facing up (a, e, and i; sequence of slices from anterior to posterior) were blocked around the macroscopically visible V1/V2 border (red boxes). Blocked samples, after labelling with methylene blue, show distinct contrast between grey and white matter (b, f, and j: anterior side of the samples; c, g, and k: posterior side of the samples). All samples are well cleared and become highly transparent, even in the white matter, after immersion in ECi (**d**, **h**, and **l**). In **d**, **h**, **l**, the grid is 1x1 mm (smallest squares).

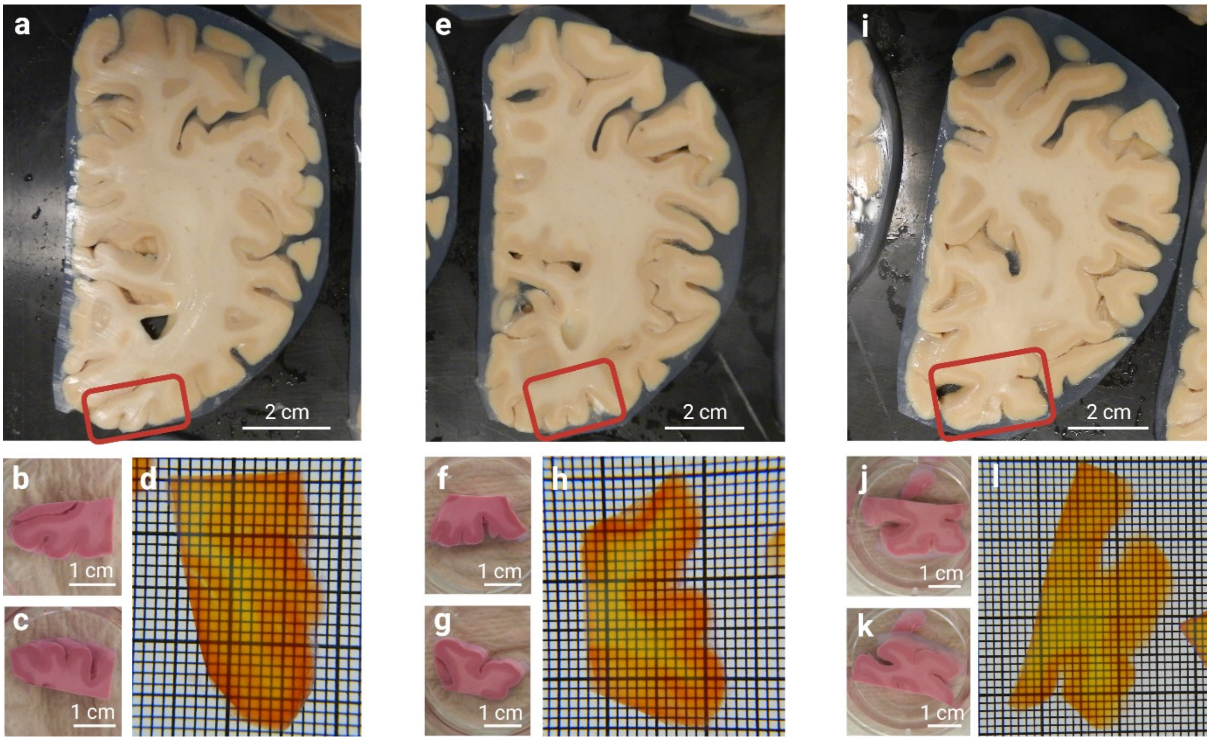

Supplementary Figure 10: Histological processing of Occipital lobe 2 samples with MASH-NR. Whole occipital lobe slices shown with the anterior side facing up (a, e, and i; sequence of slices from anterior to posterior) were blocked around the macroscopically visible V1/V2 border (red boxes). Blocked samples, after labelling with neutral red, show distinct contrast between grey and white matter (b, f, and j: anterior side of the samples; c, g, and k: posterior side of the samples). All samples are well cleared and become highly transparent, even in the white matter, after immersion in ECi (**d**, **h**, and **l**). In **d**, **h**, **l**, the grid is 1x1 mm (smallest squares).

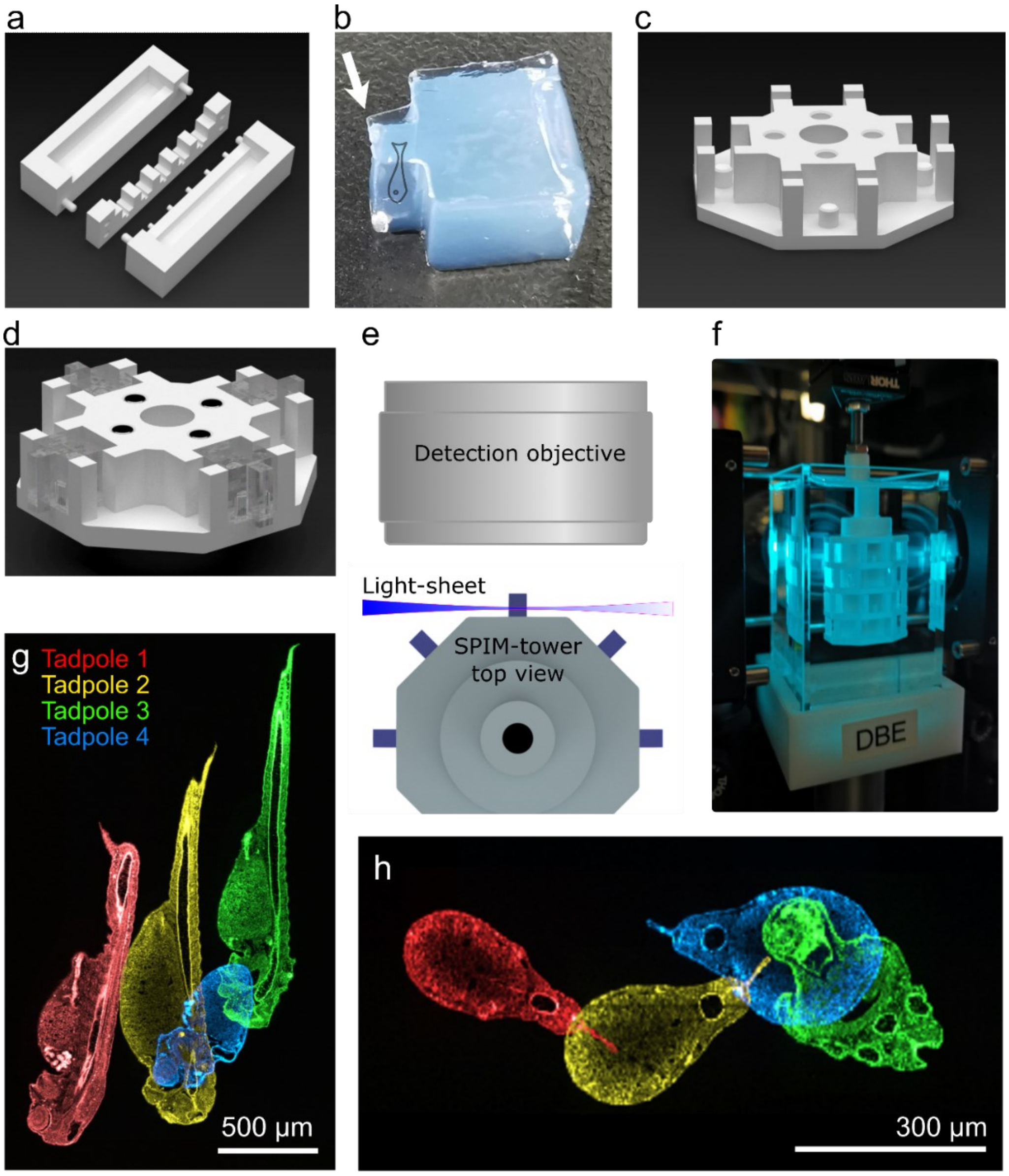

Supplementary Figure 11. The *SPIM-tower* sample holder and *SPIM-mold*: a standardized high-throughput imaging on the mesoSPIM system. **a,** CAD model of the *SPIM-mold* for reproduceable embedding of biological samples in agarose blocks. The mold has been optimized to allow for the positioning of 6 *X. tropicalis* tadpoles simultaneously in a standardized way. A pin on the *SPIM-mold* generates a negative hole for future mounting into the *SPIM-tower*. **b,** The resulting agar block with embedded sample (tadpole, position indicated by cartoon drawing). The larger part of the block is for sample mounting into the tower, and the smaller part contains the sample (arrow) and protrudes from the tower, like a small balcony. This design facilitates access of the light-sheet to the sample. **c,** One section (level) of the *SPIM-tower* holder, with 4 holes in the center for push-magnets, and 4 sample slots positioned for mounting agar blocks. Each slot has a mounting pin to fit securely into the hole in the agar block. This allows consistent and reproducible mounting of samples into the *SPIM-tower*. **d,** Four agar blocks containing samples, mounted onto one level of the *SPIM-tower*. The push-magnets allow stacking multiple levels to form a tower. **e,** Top view of the *SPIM-tower* with light-sheet illuminating the sample, and the detection objective shown. Consecutive levels are rotated by 45° from each other to avoid samples from different levels interfering with each other. **f,** The *SPIM-tower* in the Benchtop mesoSPIM during imaging. Each sample can be accessed by 90° rotation within the same level and Y translation (7.5 mm) + 45° rotation from one level to the next. **g,h** Visualization of the deviation in position of 4 stage-37 tadpoles (pseudo-colored) on one SPIM-tower level in the X-Y (**g**) and XZ (**h**) axes. Due to the *SPIM-mold* design and standardized agarose blocks, the X, Y deviations are within 1 mm and Z within 0.3 mm.

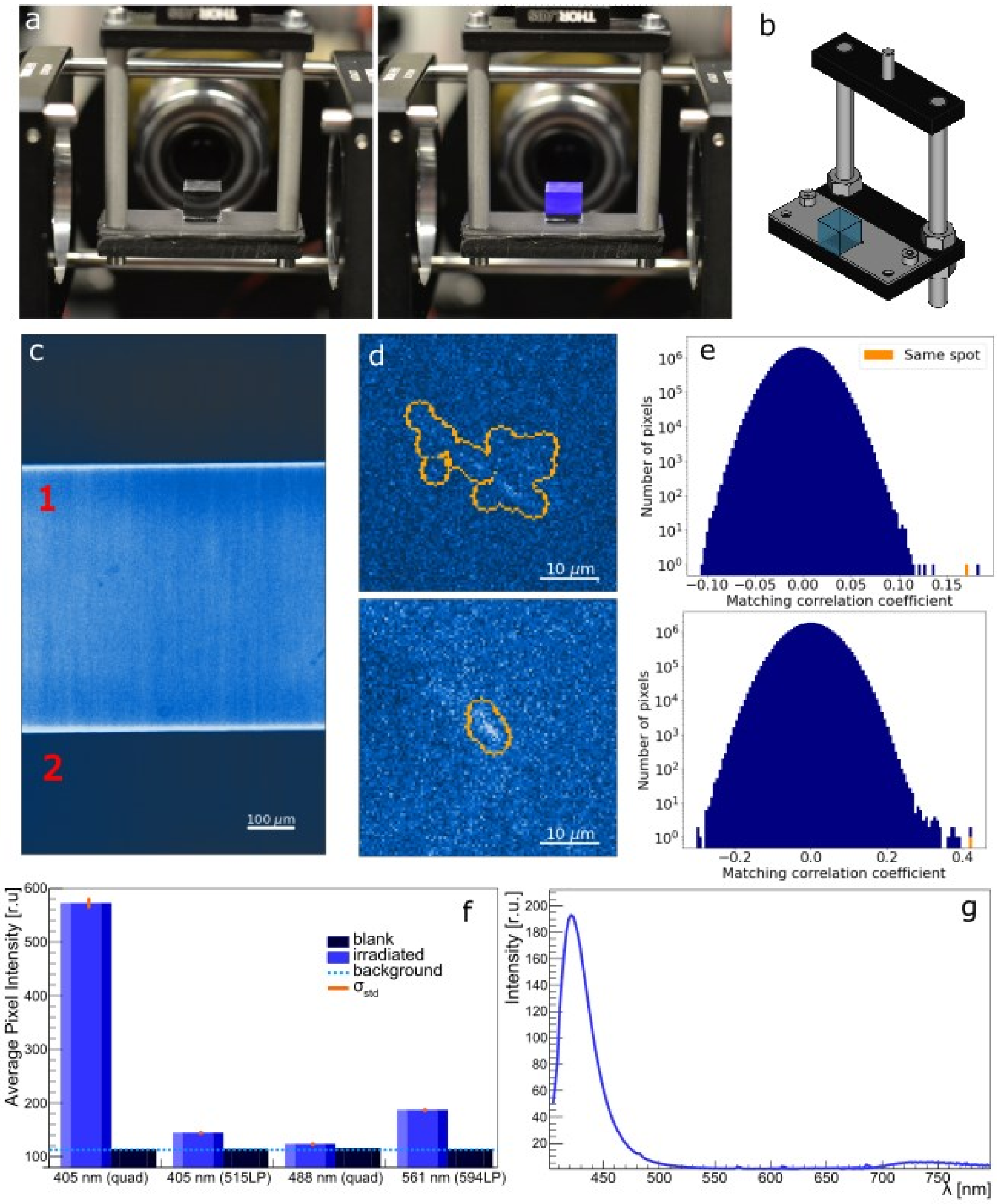

Supplementary Figure 12. Imaging color centers in CaF_2 crystal in the Benchtop mesoSPIM. The crystal was irradiated with gamma rays at 5 MRad, and imaged using Mitutoyo 20x/0.28 (t3,5) objective and 405nm excitation, without immersion. **a,** The gamma-irradiated CaF_2 crystal is highly fluorescent in the blue spectrum when illuminated with 405-nm laser (left panel: laser off, right panel: laser on). **b**, crystal sample holder design for cubic crystals, no immersion (imaging in air). **c,** Light-sheet illuminated area (bright band, ROI #1) is compared to non-illuminated areas (two dark bands, with ROI#2) for a irradiated CaF_2 crystal. **d**, Clusters of high-intensity pixels found in the scans of a CaF_2 crystal using the algorithm described in Supplementary Note 2. **e,** Matching correlation coefficients of groups of N pixels in repeated scans. The bins in orange show the matching correlation coefficients of the same group of pixels within the contours shown in the images on the left side of each graph. See Supplementary Note 2 for a detailed description. **f,** Quantification of crystal fluorescence from blank and irradiated (100 kRad) CaF_2 crystals imaged at 1x with different excitation laser wavelengths and emission filters. The background line represents the camera noise measured when the laser is off. The small error bars in orange show the standard deviation of the average pixel intensities from 350 z-planes. The solid bars show the mean value of the distribution. **g,** fluorescence spectra from the same irradiated CaF_2 crystal measured in response to 400 ± 10 nm excitation light. Abbreviation: r.u., relative units. Source data for the graphs is provided as Source Data files.

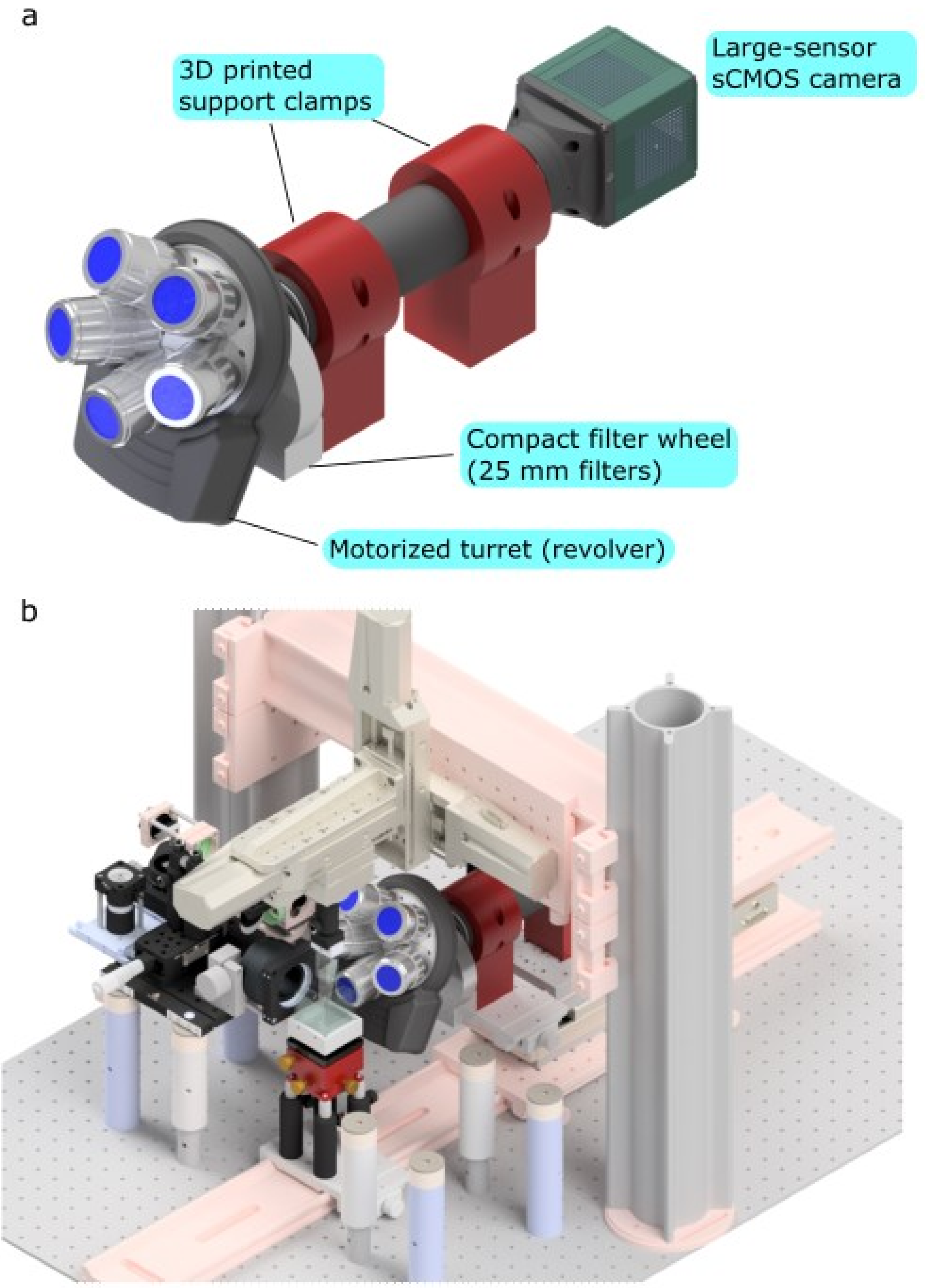

Supplementary Figure 13. CAD model of the new detection arm for mesoSPIM v4-5 upgrade. a, The detection arm elements. The height of support allows drop-in replacement into the existing detection path of mesoSPIM v4-5; **b,** The new detection arm in mesoSPIM v.5. The right excitation arm is invisible for presentation purposes.

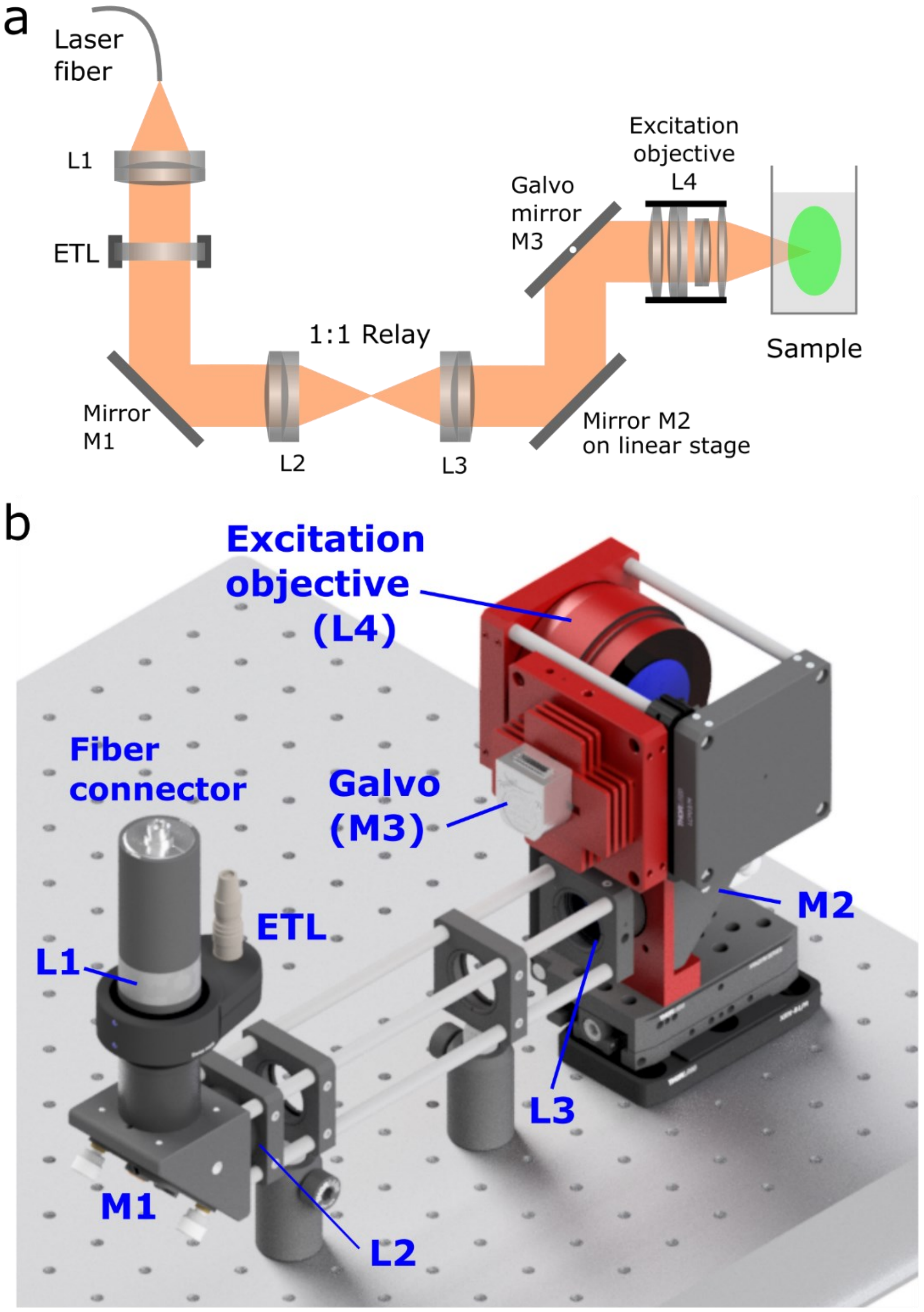

Supplementary Figure 14. The Benchtop mesoSPIM excitation arm. a, Diagram of the laser beam propagation and optical components. **b**, Mechanical implementation of the left excitation arm (view from behind). Custom-made or modified parts are color-coded in red (mirror bracket, galvo mount, galvo heat sink, Nikon 50 mm f/1.4 G and its mount). All parts and details are available at the github repository. Abbreviations: ETL, electro-tunable lens.

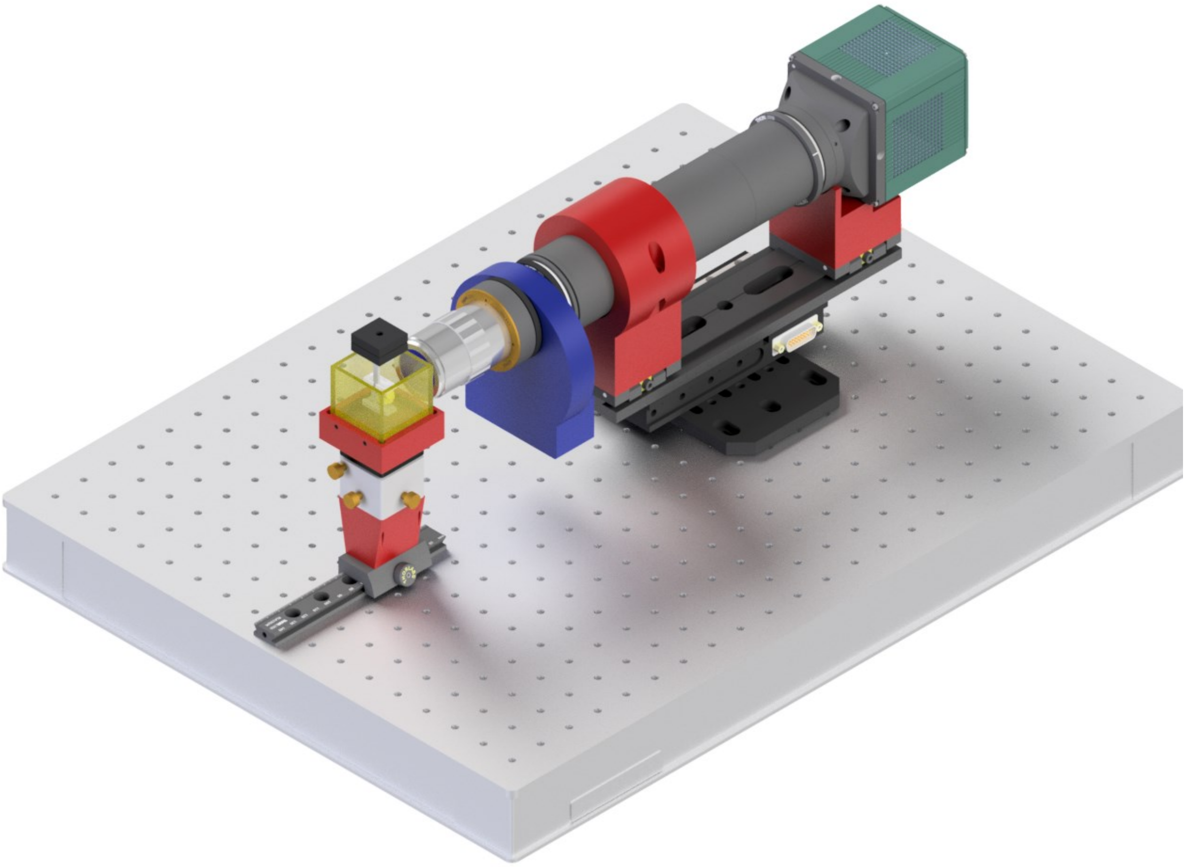

Supplementary Figure 15. Immersion chamber in the detection path of Benchtop mesoSPIM.

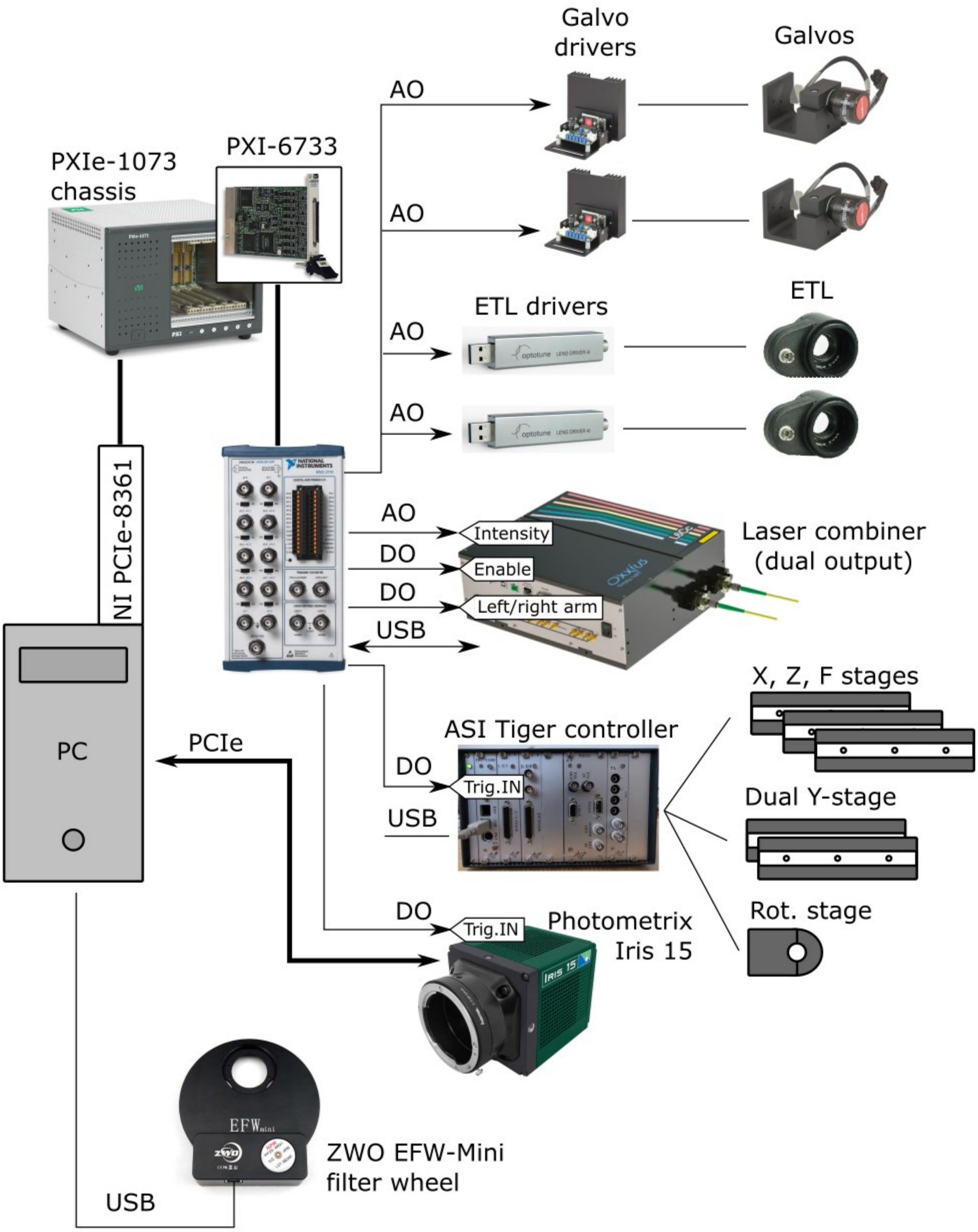

Supplementary Figure 16. Electronics block diagram of Benchtop mesoSPIM. Abbreviations: AO, analog output; DO, digital output, ETL, electro-tunable lens, Rot., rotation.

Supplementary tables

Supplementary Table 1. Properties of microscope objectives and telecentric lenses computed from their contrast maps

Cameras: Teledyne Photometrics Iris 15 (sensor dimensions Y x X: 21.49 mm x 12.61 mm) for all objectives except Olympus MVPLAPO-1x (mesoSPIM v.5, Hamamatsu Orca Flash 4.3)
Medium: air + 20 mm high-index (RI=1.52 oil);
Scanning: F-stack with f-steps 10 µm between planes.
Illumination: smartphone (white screen)
Test target: Thorlabs Ronchi ruling 40 lp/mm (R1L3S14N);
Tube lenses: Mitutoyo MT-1 for Mitutoyo objectives, Olympus wide-field tube lens SWTLU-C for Olympus 4x/0.28 and Thorlabs objectives, Olympus MVX-TLU for Olympus MVPLAPO-1x (with zoom module) objective.
Field size, mm: FOV_Y = 21.49/M, FOV_X = 12.61/M for Teledyne Photometrics Iris 15 experiments, FOV_Y = FOV_X = 13.312/M for Hamamatsu Orca Flash 4.3 experiments, where M is the effective magnification of the objective.

**Objective**	**Field sag over FOV_Y (long axis), µm**	**Field sag over FOV_X (short axis), µm**	**Max contrast in the best-focus plane**	**Ave contrast in best-focus plane**	**Min contrast in the best-focus plane**
**PlatinumTL™ 0.9x Telecentric Lens**	21	20	0.49	0.45	0.37
**Lensagon T25M-12-155I Telecentric Lens**	42	5	0.63	0.58	0.45
**Mitutoyo BD Plan Apo 2x/0.055**	90	22	0.75	0.65	0.45
**Mitutoyo BD Plan Apo 5x/0.14**	18	10	**0.86**	**0.82**	**0.65**
**Mitutoyo BD Plan Apo 7.5/0.21**	**4**	**4**	0.60	0.58	0.53
**Mitutoyo BD Plan Apo 10/0.28**	**< 1**	**4**	0.39	0.37	0.35
**Mitutoyo G Plan Apo 20x/0.28(t3,5)**	**1**	**2**	0.48	0.43	0.37
**Olympus MVPLAPO-1x, zoom 1x**	50	70	0.57	0.48	0.34
**Olympus MVPLAPO-1x, zoom 1.25x**	22	11	0.70	0.54	0.14
**Olympus MVPLAPO-1x, zoom 2x**	175	36	0.84	0.52	0.11
**Olympus MVPLAPO-1x, zoom 4x**	28	20	0.60	0.49	0.35
**Olympus MVPLAPO-1x, zoom 5x**	19	15	0.55	0.47	0.29
**Olympus MVPLAPO-1x, zoom 6.3x**	13	12	0.53	0.42	0.25
**Thorlabs Super Apochromatic 2x/0.1 TL2X-SAP**	51	13	0.72	0.62	0.43
**Thorlabs Super Apochromatic 4x/0.2 TL2X-SAP**	39	11	0.62	0.55	0.44
**Olympus XLFluor 4x/0.28**	26	15	0.41	0.33	0.18

Supplementary Table 2. Currently recommended objectives for Benchtop mesoSPIM.

The table below is based on the lenses that we have tested and should serve only as a starting guide. It is neither final nor complete compilation of possible options.

Model	Mag/NA	WD, mm	Resolution, µm	Max image circle (diagonal), mm	Approx cost, USD	Notes
**PlatinumTL™ 0.9x Telecentric Lens**	0.9x/0.045	111	5.5	28.7	2500	Telecentric lens*
**Lensagon T25M-12-155I**	1.2x/0.08	155	4.2	32	6700	Telecentric lens*
**Mitutoyo (M or BD) Plan Apo**	2x/0.055	34	5	30	1100	Mitutoyo lens**. Field flatness acceptable.
**Mitutoyo (M or BD) Plan Apo**	5x/0.14	34	2	30	900	Mitutoyo lens**. Field flatness excellent. Spherical aberration minimal even in thick samples.
**Mitutoyo (M or BD) Plan Apo**	7.5/0.21	34	1.3	30	1800	Mitutoyo lens**. Field flatness excellent. Spherical aberration acceptable for samples up to 10 mm thick.
**Mitutoyo (M or BD) Plan Apo**	10/0.28	34	1.0	30	1200	Mitutoyo lens**. Field flatness excellent. Spherical aberration acceptable for samples up to 5 mm thick.
**Mitutoyo G Plan Apo 20x(t3,5)**	20/0.28	29.42	1.0	30	3600	Mitutoyo lens**. Field flatness excellent. Spherical aberration corrected for samples up to 3.5 mm thick (n1.52).

* F-mount directly to the camera. Filter wheel (2”-filters) in front of the lens. Galvos require active (fan) cooling.
** Requires Mitutoyo MT-1 (f=200m) or similar large-FOV tube lens. Filter wheel (1”-filters) behind the objective (infinity space). The BD version has larger diameter and mounting thread and requires 3D printed screen ring.

Supplementary Table 3. Samples, labelling, clearing, and acquisition parameters.

Figure / movie	Organism, line	Staining and clearing	Excitation**	Detection*	Notes
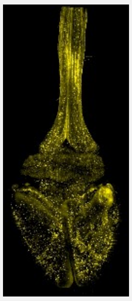<br>Figure 2, 4<br>Supplementary Movie 2.	Mouse brain & spinal cord dissected from a 2.5-month old male Thy1-GFP line M animal.	Nanobody anti-GFP conjugated to Atto647n: GFP-Booster Atto647N (ChromoTek Cat# gba647n-100).<br>Propidium iodide.<br>vDISCO clearing.	638 nm<br>z_step = 3 µm.	Mitutoyo BD Plan Apo 5X/0.14	19 tiles/ch, 779 GB.
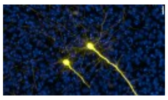<br>Figure 5	Same as above	Same as above	561, 638 nm<br>z_step = 2 µm.	Mitutoyo G Plan Apo 20X/0.28(t3,5)	1 tile/ch.
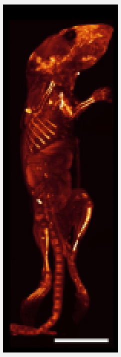<br>Figure 5,<br>Supplementary Movie 3	Whole body of a mouse pup (P14), CX3CR1 line.	Propidium iodide staining (561 excitation), nanobooster Atto647 for immune cells (microglia cells in mouse brain)<br>vDISCO whole-body staining	561 nm,<br>Scanning: f_galvo = 49.99 Hz<br>z-step = 15 µm<br>galvo_amp = 3.0 V	PlatinumTL™ 0.9x/0.045 telecentric lens	6 tiles/ch, dual illumination, 233 GB. Active (fan) cooling of galvos to avoid overheating.<br>Imaging only with the moving cuvette (without the stationary cuvette), filed with DBE, by using focus tracking and ETL parameter adjustment depending on cuvette position.
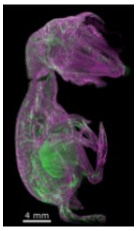<br>Figure 5,<br>Supplementary Movie 4	Chicken embryo with neurofilament staining at E9 (#4543)	Neurofilament staining with M Anti-RMO270, G Anti-Mouse Cy3<br>BABB clearing	488 nm (auto-fluorescence), 561 nm (neurofilament)<br>z_step = 5 µm	Lensagon 1.2x/0.08 telecentric lens	2 tile/ch, 230 GB total.
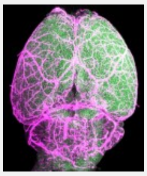<br>Figure 5,<br>Supplementary Movie 5	Mouse brain, APPPS-1 mouse	Staining: hFTAA (amyloid plaques)<br>SMA-Cy3 (arterial vessels)<br>iDISCO clearing	488 nm (hFTAA),<br>561 nm (SMA-Cy3)<br>z_step = 3 µm	Lensagon 1.2x/0.08 telecentric lens	1 tile/ch, 136 GB total.

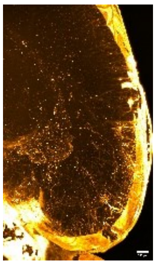<br>Figure 5,<br>Supplementary Movie 6	Mouse brain, Vglut2-Cre mouse	Retrograde AAV cre-dependent variant of td-Tomato (ssAAV-retro/2-CAG-dlox-tdTomato (rev)-dlox-WPRE - bGHp(A)<br>iDISCO clearing	561 nm<br>z_step = 2 µm	Olympus XLFluor-340, 4x/0.28	1 tile/ch, 70 GB total.
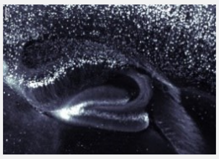<br>Supplementary Movie 7	Mouse brain, hippocampus region	Thy1-YFP AF-561<br>iDISCO clearing	561 nm<br>z_step = 2 µm	Mitutoyo BD Plan Apo 5X/0.14	1 tile/ch, 65 GB total. Sample immersed in moving cuvette (5x10x45 mm) filled with DBE, dipped into a stationary chamber (40x40x50 mm) filled with DBE.
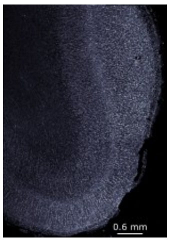<br>Figure 5,<br>Supplementary Movie 8	Human cortex, occipital lobe 2 (90-year-old male)	Staining Neutral Red.<br>Clearing with MASH-NR (ECi) protocol.	561 nm<br>z_step = 5 µm	Mitutoyo BD Plan Apo 5X/0.14	6 tiles/ch, 129 GB total. Sample immersed in dipping cuvette (10x20x45 mm) filled with ECi, dipped into a stationary chamber (40x40x75 mm) filled with DBE.
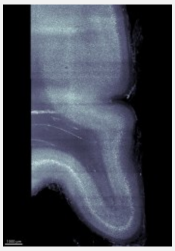<br>Supplementary Movie 9	Human cortex, occipital lobe 1 (101-year-old female)	Staining with Methylene Blue/ Clearing with MASH-MB (ECi) protocol.	638 nm<br>z_step = 5 µm	Mitutoyo BD Plan Apo 5X/0.14	12 tiles/ch, 110 GB total, clamped and dipped into a stationary chamber (40x40x50 mm) filled with ECi.
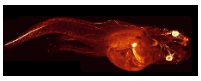<br>Supplementary Movie 10.	Xenopus tadpole stage 58 (44 days post-fertilization)	Primary AB: Atp1a1, secondary: Alexa-Fluor-555 goat anti-mouse IgG.<br>BABB clearing	561 nm<br>z_step = 7 µm	Mitutoyo BD Plan Apo 5X/0.14	40 tiles/ch, 1.68 TB, sample immersed in moving cuvette (10x10x45 mm), dipped in stationary cuvette (40x40x75 mm), both filled with DBE.

For all datasets, stitching and fusion was performed in Fiji/BigStitcher, visualization in Imaris.

* Detection filter: QuadLine Rejectionband ZET405/488/561/640.
** Default scanning parameters: *t_exp* = 20 ms, *f_galvo* = 99.99 Hz, *galvo_amp* up to 3 V depending on FOV size.

Supplementary Table 4. sCMOS cameras supported by the mesoSPIM control software.

The camera needs programmable rolling shutter readout (“light-sheet”) mode.

Model	FOV: W x H, mm	Sensor diagonal, mm	Pixel array	Pixel size, µm	Mega-pixels	Status	Notes	mesoSPIM version
**Hamamatsu Orca 4.0v3**	13.312 x 13.312	18.8	2048 x 2048	6.5	4.2 MP	Supported and recommended	Use the version with PCIe frame grabber, not the USB3 (the latter is too slow)	v4-5
**Hamamatsu Orca Fusion**	14.976 x 14.976	21.2	2304 x 2304	6.5	5.3 MP	Tested, stable	Use CoaXPress frame grabber version, NOT the USB3.	v4-5
**Hamamatsu Orca Lightning**	25.344 x 14.256	**29.1**	4608 x 2592	5.5	12 MP	Tested, stable	High resolution across entire FOV only with large-FOV detection objectives.	V4-5 upgrade option
**Teledyne Photometrics Kinetix**	20.8 x 20.8	**29.4**	3200 x 3200	6.5	10.2 MP	Tested, stable	High resolution across entire FOV only with large-FOV detection objectives.	v4-5
**Teledyne Photometrics Prime BSI**	13.312 x 13.312	18.8	2048 x 2048	6.5	4.2 MP	Tested, use at your own risk		v4-5
**Teledyne Photometrics Iris 15**	21.49 x 12.61	**24.9**	5056 x 2960	4.25	15 MP	Supported and recommended	High resolution across entire FOV only with large-FOV detection objectives.	Benchtop and v4-5 upgrade

Supplementary Table 5. Stages and motion controllers supported by the mesoSPIM

Controller	Stages	Notes	mesoSPIM version
**PI C-884**	L-509.20DG10, L-509.40DG10, M-060.DG, M-406.4PD	Serves up to 6 stages, including rotation.	v4-5 default controller
**PI C-663**	Any of the PI stages above	Serves 1 stage, multiple controllers can be used.	v5 option
**ASI Tiger (TG8-BASIC)**	LS-50, LS-100, C60-3060	Serves 6 motors with 3 additional free slots.	Benchtop

Abbreviations: PI, Physik Instrumente; ASI, Applied Scientific Imaging.

Supplementary Table 6. Filter wheels supported by the mesoSPIM

Model	Max. filter diameter, mm	N(filter slots)	Notes	mesoSPIM versions
**ZWO EFW-MINI**	31 mm	5	Very compact and low-cost astronomy filter wheel, with integrated controller and USB cable.	Benchtop
**Ludl 96A350**	32 mm	10	Large filter wheel with separate controller (MAC6000), serial cable, and high cost.	v4-5
**Sutter Lambda 10**	25	10	deprecated	Used in early versions.

Supplementary Table 7. Parts list for mesoSPIM v4-5 upgrade

All prices are approximate and subject to change at any time by the vendors.

Part	Qty	Cost/unit, USD
Mitutoyo M Plan Apo 2x/0.055	1	1,100
Mitutoyo M Plan Apo 5x/0.14	1	900
Mitutoyo M Plan Apo 7.5x/0.21	1	1,800
Mitutoyo M Plan Apo 10x/0.28	1	1,200
Mitutoyo G Plan Apo 20x(t3,5)	1	3,600
Mitutoyo MT-1 tube lens	1	900
Mitutoyo 378-726D motorized turret (revolver)	1	4,600
Photometrics Iris 15 camera	1	12,000
Other hardware	-	800
Total, USD		26,900

The detection filters as well as focusing and sample stages can be re-used.

Supplementary notes

Supplementary Note 1: Acquisition speed and file size

To image 1 cm^3 (dimensions S_x = S_y = S_z = 10 mm) of sample in multi-tiling acquisition, the number of frames along each dimension are

$$N_x = \frac{S_x}{FOV_x\, P_{overlap}}, \qquad N_y = \frac{S_y}{FOV_y\, P_{overlap}}, \qquad N_z = S_z/\Delta z \qquad (1)$$

where FOV$_x$ and FOV$_y$ are field of view dimensions on the sample side (camera chip dimensions divided by the system magnification), $P_{overlap}$ = 0.9 is the tile overlap ratio in X and Y used for stitching (10%, default setting), Δz is the z-step between planes.
The total acquisition time is

$$T_{acq} = N_x N_y N_z / FPS \qquad (2)$$

where FPS is the effective frame rate (frames/s).
Fast mode. In this mode one can choose under-sampling in Z while still maintaining sufficient resolution for axons and dendrites detection.
Nyquist mode. This mode samples the axial PSF of the system at the optimal step, about 2 planes per PSF axial size, FWHM$_z$.

Supplementary Table 8. Estimated acquisition time and file size for imaging 1 cm^3 sample on the Benchtop mesoSPIM

Camera: Teledyne Photometrics Iris 15. Acquisition frame rate of 2.5 images per second includes data writing time on disk. Time and size are estimated per channel, averaged per sample volume. Actual times may vary due to integer number of tiles covering the sample volume.

	**Fast mode** (z-step 5 μm)		**Nyquist mode** (z-step 2 μm)	
**Magnification**	Minutes, per 1 cm^3 actual**, average by vol.	File size, GB actual, average by vol.	Minutes, per 1 cm^3 actual, average by vol.	File size, GB actual, average by vol.
1x	13, 6	59, 27	33, 15	149, 68
2x	53, 24	239, 109	133, 60	598, 272
5x*	200, 152	897, 681	500, 380	2244, 1704
7.5x	373, 342	1'676, 1534	933, 854	4190, 3835
10x	720, 607	3232, 2727	1800, 1518	8081, 6818
20x	2640, 2430	11'852, 10908	6600, 6074	29632, 27272

* For comparison, imaging at this magnification with mesoSPIM v.5 (camera Hamamatsu Orca Flash 4.3) in fast mode takes 333 instead of 200 min and produces 419 instead of 897 GB of data.
** Actual time is larger than (volume) average time because the the number of tiles in actual acquisition is always integer, which may be non-optimal for small volumes. At high magnifications and/or large volumes the difference between the two estimates becomes negligible.

The code for calculating the acquisition speed and file size is available as Python notebook: https://github.com/mesoSPIM/image-processing/blob/main/notebooks/volumetric-speed-calculation.ipynb

Supplementary Note 2: Imaging of color centers for particle detectors

In the main text, we present a Benchtop mesoSPIM application for imaging image color centers induced in crystals by irradiation. This application has the ultimate goal of dark matter and neutrino detection, which are relevant topics in the fields of particle physics, nuclear engineering and nuclear non-proliferation safeguards.

The objective of this section is to provide the reader outside of these fields with: 1) the background on the search and detection of elusive particles, such as dark matter and neutrinos, as well as the application of neutrino detectors as nuclear non-proliferation safeguards, 2) additional information on the topic of microscopy imaging of color centers (and its use for particle detectors), 3) an overview the current research and ongoing developments, as well as future prospects.

Color centers signals from particle interactions

Dark matter

There is clear evidence for the existence of dark matter[1,2] -- a type of matter that exerts a gravitational pull in our galaxy (and universe), but is 'dark' because it does not interact with photons in the same way ordinary matter does. Although five times more abundant than ordinary matter[1], dark matter particles have not yet been detected. Many theories predict it to be a weakly interacting massive particle. As our solar system is immersed in a dark matter sea and travels across it at 220 km/s, we should constantly receive a large flux from these particles. Although most of these particles should travel across the Earth unnoticed, we expect them to interact occasionally with the nuclei of ordinary atoms, making them recoil. Many experiments have been searching for signals from these dark matter collisions with nuclei[3–9]. Most of them rely on the collection of charge, prompt scintillation photons or phonons as dark-matter induced signals. In this work, we present the first tests done with the Benchtop mesoSPIM using a new approach, proposed by Cogswell et al.[10]: the use of SPIM to detect color centers signals, induced by the collision of particles, such as dark matter, with atoms in the lattice of a crystal.

A schematic of how dark matter (and other particles) could create color centers in a crystal is shown in Supplementary Figure 17. For a detailed review of the concept, we refer the reader to Refs[10–12]. For a detailed description of color centers, we refer the reader to Ref.[13]

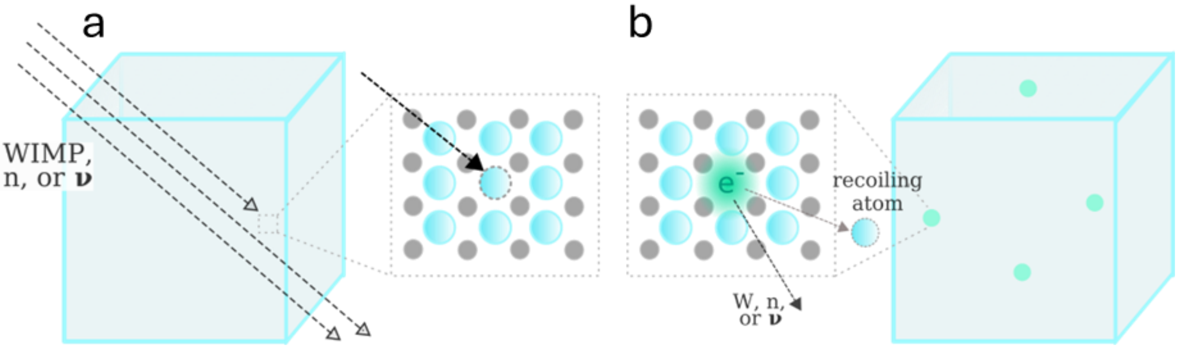

Supplementary Figure 17: **a,** A transparent crystal is exposed to a flux from particles: dark matter (Weakly Interacting Massive Particle, WIMP), neutrons (n) or neutrinos (ν). If one of these particles interacts (collides) with a nucleus, the atom can recoil and be permanently displaced from its lattice position. In the case of an anion, the vacancy can be occupied by an electron, forming an F-center type of color center[14]. **b,** This center appears as a fluorescent point in a SPIM image of the crystal. For more massive/interacting particles, such as cosmic-rays, multiple atoms can be displaced forming a *track* of color centers. For gamma-rays, no atom displacement is expected, but ionization and consequent

charge redistribution can also create color centers of different types, as described in Ref[14]. After exposure, the color centers are imaged with SPIM and their position and intensity are recorded.

Nuclear reactor neutrinos

To detect another elusive particle, the neutrino, ton-sized detectors or powerful accelerators are usually required to obtain a few events per day[15–17]. While elastic collisions of neutrinos with nuclei have just recently been observed using accelerator neutrino sources[16], these interactions from reactor neutrinos have not yet been detected. The challenge is to bring a very sensitive detector close enough to the reactor. Small passive detectors, such as transparent crystals, could be more easily placed close to the reactor and work as nuclear-nonproliferation safeguards[10]. In this application, crystals would be exposed to the high flux of neutrinos from the reactor and scanned with SPIM (*ex-situ*) to image neutrino-induced color centers. By counting the number of new centers in the crystal, it would be possible to understand the reactor power[10]. This knowledge could be then used to estimate (or exclude) the production of plutonium in the monitored reactor[10].

Imaging of color centers

While the interaction of elusive particles (W, ν) with the crystal lattice cause atom dislocations of only a few nanometers (Supplementary Figure 17), color centers can be probed with fluorescence microscopy at optical wavelengths. The fluorescence response of color centers enables faster and less expensive imaging than other techniques used to probe nm-sized features, such as transmission electron microscopy (TEM) and atomic force microscopy (AFM).

The imaging of single color centers has already been performed with widefield and confocal fluorescence microscopes[18,19]. Imaging these color centers with SPIM offers further advantages: good optical sectioning compared to widefield imaging, less bleaching, higher throughput, and larger maximum sample size compared to confocal imaging.

The non-destructive, fast and isotropic scan of large 3D-volumes offered by mesoSPIM is thus well suited for imaging large amounts of color-center based particle detectors. Another possible application of color center imaging with the mesoSPIM is fission track dating of transparent rocks: the mesoSPIM could offer a fast imaging method for large amounts of minerals without the need of etching, as described in Ref[12].

Benchtop mesoSPIM imaging of particle-induced color centers in CaF_2

In this work, we show the first tests of imaging particle-induced color centers with the mesoSPIM. This is part of the international collaborative work of PALEOCCENE (Passive low-energy optical color center nuclear recoil) – a group of scientists working on the R&D of passive detectors of dark matter and neutrinos at low energy thresholds[11].

For the purpose of initial tests, we acquired transparent CaF_2 crystals, irradiated them, and imaged them with the mesoSPIM, as described in the Methods section. The Supplementary Figure 12a shows the crystal ready for imaging before and while the light sheet is on. A custom-made crystal holder, shown in Figure 12b was made for keeping the samples in a stable position and allowing for changing crystals in consecutive scans without the need of complete refocusing. The fluorescence of an irradiated crystal in response to the 405 nm light sheet is clearly distinguished from the background, as shown by the contrast of the illuminated area in Figure 12c. While the fluorescence is mostly homogeneous across the crystal (presumably from many nm-sized color centers), a few clusters of high-intensity pixels appear at small scales (~10 µm). These structures are especially clear when imaged with the 20x/0.28 objective. A few examples are shown in Figure 12d.

The structures shown in Supplementary Figure 12d are identified by an algorithm, which scans the area of the illuminated ROI and outputs the contours of clusters of high-intensity pixels. The parameters used to define the selection thresholds of intensity and clustering are preliminary and will be benchmarked and improved in the future with data from dedicated ion-irradiation (track-inducing) campaigns.

To verify whether the selected structures are intrinsic to the crystal – and not some random noise from the camera – repeated scans are compared. The matching correlation between them is calculated with the `skimage.feature.template_matching` method, where the smallest rectangle enclosing the feature with N pixels is compared to the repeated scan using a normalized cross-correlation. The result ranges from -1 to 1, where 1 represents a perfect match. Most of the values are around zero, which is expected from random matching of any given group of N pixels which do not contain structures. The first two examples of the identified structures shown in Supplementary Figure 12d-e are observed at the same respective spots in the repeated scans and thus display large matching correlation coefficients (shown in red in e). Their matching coefficients are 7.9 and 7.3 sigma away from the mean of their respective distributions. The other values similar to the ones in red are due to the matching with neighboring z-planes, as the imaged structures span across a few z-scans. These distributions vary as the N number of pixels in the rectangles also vary.

While this analysis shows that these "track-like" structures are intrinsic fluorescent features in the crystal, the origin of these features is not yet clear. One possibility is that the passage of particles such as cosmic rays may have created a track of color centers. Cosmic rays hit the Earth constantly, especially at high altitudes. The crystals were likely exposed to this natural irradiation since their production and more intensively while they were mailed by air after the purchase and for the irradiation campaigns. Despite the unknown origin of these features, this study demonstrates the capability of the benchtop mesoSPIM in identifying "track-like" structures of color centers. While the tracks from dark matter or neutrino interactions would have sizes below the resolution power of the mesoSPIM, understanding track formation is part of the R&D process of the concept. Furthermore, "the energy reconstruction of dark matter / neutrino interactions may be possible through the larger intensity of fluorescence (pixel brightness) measured from a region containing a full track in comparison to a single-site color center"[12]. The improved resolution provided at 20x magnification will be especially relevant when imaging single color centers or single tracks from nuclear recoils produced by neutrons – a proof-of-concept test planned as a next step.

With the mesoSPIM images of the crystals irradiated with gamma rays, we performed further analyses with the aim of understanding: i) the distribution/homogeneity of color centers at larger scales (across milliliters of material), ii) the sources of background, and iii) the color of the imaged color centers, and how the results obtained with the mesoSPIM compare to far-field spectroscopy.

To quantify the level and homogeneity of fluorescence from blank and irradiated crystals, we imaged them at 1x magnification and calculated the average pixel intensity from every z-image of 350 scans taken at 10 µm steps inside the crystals. The obtained mean values and the respective gaussian standard deviations are shown in Supplementary Figure 12f. For this estimation, only pixels inside the illumination ROI #1 and at a distance larger than ~0.2 mm from the surface were selected to avoid surface background (as we found that the surface of blank crystals present a slightly higher signal intensity than their bulk). Possible sources of the higher surface fluorescents include dust or any other residual material which is auto-fluorescent and difficult to remove by usual cleaning methods, such as machine oils or surface coating, depending on the manufacturing of the crystal. The bulk of *blank* (not irradiated or before irradiation) crystals displayed low net fluorescence level, as observed in Supplementary Figure 12f: the fluorescence intensities in response to all the selected laser excitations were very close to the background level, which was estimated with the laser off.

By imaging these crystals in the mesoSPIM with different laser excitations (405, 488, and 561 nm) and filters (quadrupole or long pass), we can understand the colors / wavelengths absorbed and reemitted as well as verify whether the signal corresponds to fluorescence or Raman scattering. Supplementary Figure 12f shows that irradiated crystals absorb 405 nm light and fluoresce in the blue: the most intense emission is in response to 405 nm light and faints when the blue spectrum is cut by a 515 nm long-pass filter. This blue fluorescence has been confirmed by the absorption and emission spectra measured in response to light from 250 to 800 nm with an Edinburgh Instruments FS5 spectrofluorometer. An example of the measured fluorescence from an irradiated crystal in response to 400 ± 10 nm light is shown

in Supplementary Figure 12g. The response to shorter excitation wavelengths (down to ~340 nm) consistently presents the emission peak at ~ 425 nm. This result validates the observations obtained with the mesoSPIM: the irradiated crystals fluoresce in blue and the signal is not Raman scattering. A fainter emission peak at ~740 nm is also observed and its intensity increases at larger excitation wavelengths (~600 nm). This also matches the results obtained with the mesoSPIM: a significant fluorescent signal in response to 561-nm excitation light.
While the mesoSPIM data agrees with the far-field spectroscopy, there are several advantages offered by the mesoSPIM. With the sectioned images, we can define an ROI avoiding surface background, estimate the fluorescence colors as well as quantify the distribution of color centers across the sample. The mesoSPIM can also image single clusters of color centers and precisely identify their position, a feature that is crucial for the concept of particle detectors discussed in this section.

Supplementary Note 3: Effects of spherical aberration on excitation beam

The presence of a thick (10-30 mm) layer of immersion medium is expected to broaden the excitation beam and the detection PSF due to spherical aberration. To estimate these effects, we simulated a simplified system in OptalixPro software (Supplementary Figure 18), with excitation objective simulated as ideal lens (f=50 mm) and with chamber glass walls neglected for simplicity (their refractive index 1.52 is close to the imaging medium 1.56). From the Supplementary Table 9 one can see that the beam diameter slowly increases with medium thickness up to 20 mm, then transiently decreases at thickness 30 mm, and then increases again at thickness 40 mm. In practical mesoSPIM usage, the beam is expected to remain in the range between 2.4 and 3.0 µm (medium thickness 10-30 mm).

In the absence of ASLM scanning (ETL amplitude set to 0) we experimentally measured the beam waist diameter FWHM to be 2.8±0.33 µm (mean±std, n=103 beads) for the 488 nm excitation, which agrees almost perfectly with the simulations. The experimental results are available at notebook https://github.com/mesoSPIM/mesoSPIM-PSFanalysis/benchtop-MitutoyoBD-5X(TL-MT1)-ETL-off.ipynb.

Note that in the ASLM-on mode the beam is scanned axially, so that the beam experiences a range of medium thicknesses during scanning, which effectively makes it broader, in agreement with our experimental measurements of beads in ASLM regime, which show field-averaged PSF FWHM(z) in the range of 3.3-4.0 µm (**Supplementary Figure 1**), a spread factor of about 37% compared to the ASLM-off simulated and experimentally measured beam diameter.

These simulations suggest that Nikon 50 mm f/1.4 G excitation objectives achieve close to diffraction limited performance for NA=0.15 beam focusing, and that broadening of the excitation beam is mostly due to the immersion medium.

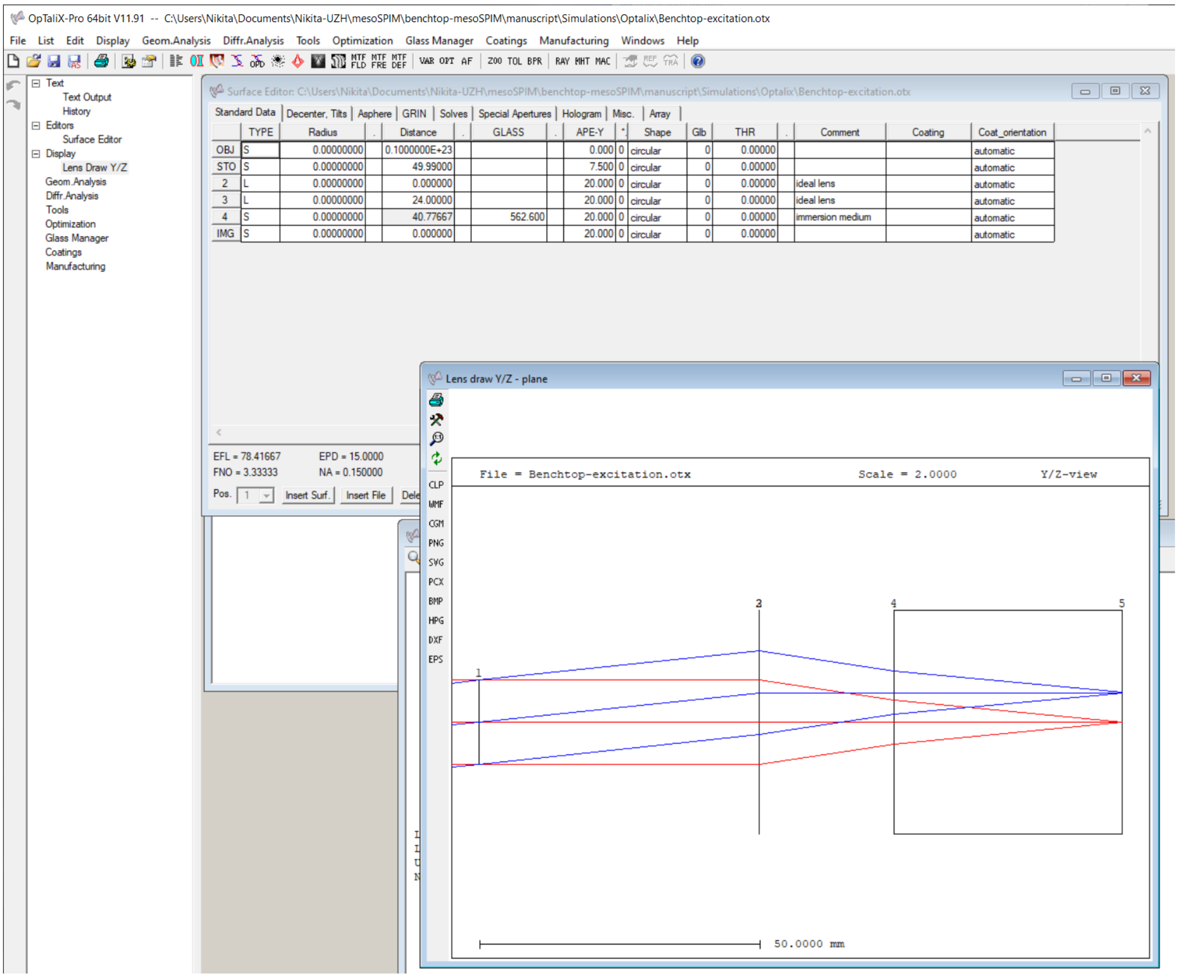

Supplementary Figure 18. Simplified model of the excitation objective and immersion chamber. The surface #4 is where the immersion medium (refractive index 1.562) starts. Surface #5 is the plane where beam profile PSF is evaluated. Apodization was set to 0.135 to mimic the Gaussian beam.

Supplementary Table 9: Simulated excitation beam waist diameter (FWHM) as a function of imaging medium thickness

Beam NA=0.15, λ=0.488 μm, medium model n=1.562@488 ("DBE"). Diffraction-limited cases (wavefront RMS < λ/4) marked in **bold**.

**"DBE" thickness**	**Beam waist FWHM (cross-section), μm**	**Spherical aberration and defocus (3-rd order) Zernike term, waves**	**Total wavefront RMS, waves**
0 mm (air)	**1.8 μm**	**0**	**0**
5 mm	**1.9 μm**	**-0.04 λ**	**0.07 λ**
10 mm	**2.4 μm**	**-0.08 λ**	**0.14 λ**
15 mm	**3.2 μm**	**-0.12 λ**	**0.21 λ**
20 mm	3.6 μm	-0.16 λ	0.29 λ
30 mm	3.0 μm	-0.23 λ	0.43 λ
40 mm	4.0 μm	-0.31 λ	0.57 λ

Supplementary Note 4: Effects of spherical aberration on detection performance

To evaluate the effect of spherical aberration (caused by the immersion medium) on the *detection* performance we simulated a simplified system: a 20-mm thick medium with a given R.I., an ideal 5x objective (f=40 mm) and an ideal tube lens (f=200 mm). The performance was measured by PSF properties such as FWHM(x,y) and Strehl ratio. While FWHM is often used to estimate the resolution, Strehl ratio estimates how much PSF intensity peak is *reduced* compared to an ideal aberration-free PSF, which affects the image contrast. An optical system with a Strehl ratio above 0.8 is considered diffraction-limited.

In agreement with our experimental results (Supplementary Figure 19), the NA of the simulated detection objective has a strong impact on the imaging performance, especially on the contrast. With NA changing from 0.15 to 0.30, the PSF FWHM(x,y) remained nearly constant (thus cancelling the positive effect of higher NA), while the Strehl ratio dropped from 0.65 to 0.04 (Supplementary Table 10), which negatively affects image contrast.

At a fixed medium thickness (20 mm) and objective NA (0.15), variation of medium refractive index in a range from 1.47 to 1.99 did not result any significant deterioration of either PSF size or Strehl ratio (Supplementary Table 11).

At a fixed medium R.I. and objective NA, the medium thickness itself had a strong impact on system's performance, such that Strehl ratio was nearly diffraction-limited at medium thickness 5-15 mm and deteriorated to 0.40 at thickness 30 mm (Supplementary Table 12), in agreement with our experience that image contrast drops when the sample and medium are thick, even for well cleared samples. We therefore remind the users that sample and medium thickness should be kept as small as possible for optimal results.

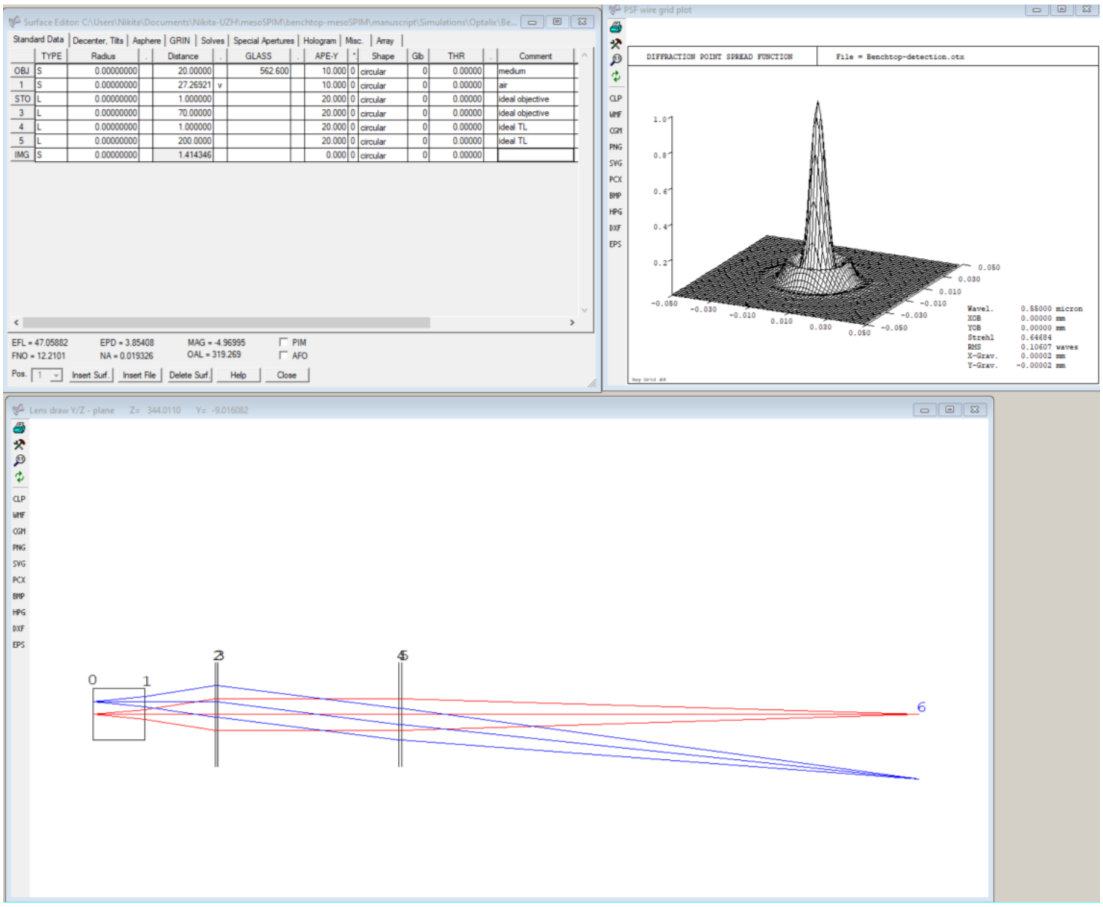

Supplementary Figure 19. Simplified model of the detection path (immersion chamber, 5x objective, and a tube lens) in OptalixPro software.

Supplementary Table 10: Simulated detection performance as a function of detection NA

"DBE" thickness set to 20 mm, R.I.=1.562, λ=0.55 μm, magnification 5x (objective f=40 mm, tube lens f=200 mm). Strehl ratio of a diffraction-limited, aberration-free PSF is 1.

Detection NA	Diffraction limited PSF size (without medium)	Aberrated PSF size, with 20 mm medium	Aberrated PSF Strehl ratio	Spherical aberration 3-rd order Zernike term, waves
**0.15**	1.9 μm	1.8 μm	0.65	-0.15 λ
**0.20**	1.4 μm	1.8 μm	0.23	-0.47 λ
**0.25**	1.1 μm	1.8 μm	0.09	-1.16 λ
**0.30**	0.9 μm	1.7 μm	0.04	-2.42 λ

Supplementary Table 11: Simulated detection performance as a function of medium refractive index.

Detection NA=0.15, medium thickness 20 mm.

Medium R.I.	Aberrated PSF size	Aberrated PSF Strehl ratio
**1.33**	1.8 μm	0.72
**1.47**	1.8 μm	0.66
**1.52**	1.8 μm	0.65
**1.56**	1.8 μm	0.65
**1.60**	1.8 μm	0.64
**1.80**	1.8 μm	0.64
**1.99**	1.8 μm	0.65

Supplementary Table 12: Simulated detection performance as a function of medium thickness

Detection NA=0.15, medium R.I.=1.56. Diffraction-limited cases (Strehl ratio >~0.80) are marked in **bold**.

Medium thickness	Aberrated PSF size	Aberrated PSF Strehl ratio
5 mm	**1.9 μm**	**0.97**
10 mm	**1.8 μm**	**0.89**
15 mm	**1.8 μm**	**0.78**
20 mm	1.8 μm	0.65
30 mm	1.6 μm	0.40

Supplementary References

1. Aghanim, N. & others. Planck 2018 results. VI. Cosmological parameters. *Astron Astrophys* **641**, A6 (2020).
2. Schumann, M. Direct Detection of WIMP Dark Matter: Concepts and Status. *J Phys G* **46**, 103003 (2019).
3. Angloher, G. & others. Results on sub-GeV Dark Matter from a 10 eV Threshold CRESST-III Silicon Detector. (2022).
4. Agnese, R. & others. Nuclear-Recoil Energy Scale in CDMS II Silicon Dark-Matter Detectors. *Nucl Instrum Meth A* **905**, 71–81 (2018).
5. Hime, A. The MiniCLEAN Dark Matter Experiment. *Proc. DPF Conf.* (2011).
6. Calvo, J. *et al.* Commissioning of the ArDM experiment at the Canfranc underground laboratory: first steps towards a tonne-scale liquid argon time projection chamber for Dark Matter searches. *J Cosmol Astropart Phys* **2017**, 003–003 (2017).
7. Agnes, P. *et al.* DarkSide-50 532-day Dark Matter Search with Low-Radioactivity Argon. *Phys Rev D* **98**, 102006 (2018).
8. Ajaj, R. *et al.* Search for dark matter with a 231-day exposure of liquid argon using DEAP-3600 at SNOLAB. *Phys Rev D* **100**, 022004 (2019).
9. Aprile, E. & others. Projected WIMP sensitivity of the XENONnT dark matter experiment. *JCAP* **11**, 031 (2020).
10. Cogswell, B. K., Goel, A. & Huber, P. Passive Low-Energy Nuclear-Recoil Detection with Color Centers. *Phys Rev Appl.* **16**, 064060 (2021).
11. Alfonso, K., Araujo, G. R., Baudis, L., Bowden, N., & others. Passive low energy nuclear recoil detection with color centers – PALEOCCENE. in *2022 Snowmass Summer Study* (2022).
12. Baum, S. *et al.* Mineral Detection of Neutrinos and Dark Matter. A Whitepaper. *Accept. Publ. Phys. Dark Universe* (2023).
13. collaboration, T. B. Comprehensive measurement of pp-chain solar neutrinos. *Nat. Vol. 562 Pages 505-510* (2018).
14. Tilley, R. J. D. *Colour and the Optical Properties of Materials, 2nd edn.* (Wiley, 2011).
15. Boudjemline, K. *et al.* The Calibration of the Sudbury Neutrino Observatory using uniformly distributed radioactive sources. *Nucl Instrum Meth* **A620**, 171–181 (2010).
16. Akimov, D. *et al.* Observation of coherent elastic neutrino-nucleus scattering. *Science* **357**, 1123–1126 (2017).
17. Agostini, M. *et al.* Comprehensive measurement of pp-chain solar neutrinos. *Nature* **562**, 505–510 (2018).
18. Ju, Z., Lin, J., Shen, S., Wu, B. & Wu, E. Preparations and applications of single color centers in diamond. *Adv. Phys. X* **6**, 1858721 (2021).
19. Sow, M. *et al.* High-throughput nitrogen-vacancy center imaging for nanodiamond photophysical characterization and pH nanosensing. *Nanoscale* **12**, 21821–21831 (2020).
