# Supplementary material for: Benchtop mesoSPIM: a next-generation open-source light-sheet microscope for cleared samples
Source: Nat Commun. 2024 Mar 27;15:2679. doi: 10.1038/s41467-024-46770-2 (PMC10973490; doi:10.1038/s41467-024-46770-2)
Supplement: Supplementary file 2 — Reporting Summary [file 41467_2024_46770_MOESM2_ESM.pdf]

nature portfolio

Corresponding author(s): N. Vladimirov

Last updated by author(s): 2024-02-29

Reporting Summary

Nature Portfolio wishes to improve the reproducibility of the work that we publish. This form provides structure for consistency and transparency in reporting. For further information on Nature Portfolio policies, see our Editorial Policies and the Editorial Policy Checklist.

Statistics

For all statistical analyses, confirm that the following items are present in the figure legend, table legend, main text, or Methods section.

n/a	Confirmed	
☐	☒	The exact sample size (*n*) for each experimental group/condition, given as a discrete number and unit of measurement
☒	☐	A statement on whether measurements were taken from distinct samples or whether the same sample was measured repeatedly
☒	☐	The statistical test(s) used AND whether they are one- or two-sided<br>*Only common tests should be described solely by name; describe more complex techniques in the Methods section.*
☒	☐	A description of all covariates tested
☒	☐	A description of any assumptions or corrections, such as tests of normality and adjustment for multiple comparisons
☐	☒	A full description of the statistical parameters including central tendency (e.g. means) or other basic estimates (e.g. regression coefficient) AND variation (e.g. standard deviation) or associated estimates of uncertainty (e.g. confidence intervals)
☒	☐	For null hypothesis testing, the test statistic (e.g. *F*, *t*, *r*) with confidence intervals, effect sizes, degrees of freedom and *P* value noted<br>*Give P values as exact values whenever suitable.*
☒	☐	For Bayesian analysis, information on the choice of priors and Markov chain Monte Carlo settings
☒	☐	For hierarchical and complex designs, identification of the appropriate level for tests and full reporting of outcomes
☒	☐	Estimates of effect sizes (e.g. Cohen's *d*, Pearson's *r*), indicating how they were calculated

Our web collection on statistics for biologists contains articles on many of the points above.

Software and code

Policy information about availability of computer code

Data collection	https://github.com/mesoSPIM/mesoSPIM-control version 1.8.1
Data analysis	https://github.com/nvladimus/lens-testing<br>https://github.com/mesoSPIM/mesoSPIM-PSFanalysis<br>ImageJ (Fiji) v 1.54c<br>Imaris v. 10.0.0

For manuscripts utilizing custom algorithms or software that are central to the research but not yet described in published literature, software must be made available to editors and reviewers. We strongly encourage code deposition in a community repository (e.g. GitHub). See the Nature Portfolio guidelines for submitting code & software for further information.

Data

Policy information about availability of data

All manuscripts must include a data availability statement. This statement should provide the following information, where applicable:
- Accession codes, unique identifiers, or web links for publicly available datasets
- A description of any restrictions on data availability
- For clinical datasets or third party data, please ensure that the statement adheres to our policy

Representative microscopy data are available in the BioStudies database (https://www.ebi.ac.uk/biostudies/) under accession number S-BIAD963.

Research involving human participants, their data, or biological material

Policy information about studies with human participants or human data. See also policy information about sex, gender (identity/presentation), and sexual orientation and race, ethnicity and racism.

Reporting on sex and gender	Sex- and gender-based analysis is not relevant in our study, because our findings are about novel microscope design and we do not make any specific biological claims. The biological sex of the donor was determined macroscopically be the anatomist during examination of the donor bodies. No statements about the donor's gender are possible, as this type of information is not standardly collected within the body donation program of the Department of Anatomy and Embryology, Maastricht University.
Reporting on race, ethnicity, or other socially relevant groupings	This study did not involve any categorization based on race, ethnicity, or other socially relevant groupings. Information on race and/or ethnicity is not available within the body donation program of the Department of Anatomy and Embryology, Maastricht University.
Population characteristics	Three human occipital lobe samples were obtained from 2 body donors each (Occipital lobe 1: 101-year-old female; Occipital lobe 2: 90 year old male; no known neurological disease, respectively).
Recruitment	The body donors were provided by the body donation program of the Department of Anatomy and Embryology, Maastricht University.
Ethics oversight	The tissue donors gave their informed and written consent to the donation of their body for teaching and research purposes as regulated by the Dutch law for the use of human remains for scientific research and education ("Wet op de Lijkbezorging"). Accordingly, a handwritten and signed codicil from each donor posed when still alive and well, is kept at the Department of Anatomy and Embryology Faculty of Health, Medicine and Life Sciences, Maastricht University, Maastricht, The Netherlands. All methods were carried out in accordance with the relevant guidelines and regulations and all experimental protocols were approved by the Ethics Review Committee Psychology and Neuroscience (ERCPN).

Note that full information on the approval of the study protocol must also be provided in the manuscript.

Field-specific reporting

Please select the one below that is the best fit for your research. If you are not sure, read the appropriate sections before making your selection.

☒ Life sciences ☐ Behavioural & social sciences ☐ Ecological, evolutionary & environmental sciences

For a reference copy of the document with all sections, see nature.com/documents/nr-reporting-summary-flat.pdf

Life sciences study design

All studies must disclose on these points even when the disclosure is negative.

Sample size	Sample size was n=1 for the mouse and chicken imaging experiments (Figures 2, 4, 5a-e), and n=16 for the tadpole imaging experiment (Fig. 5j). Sample sizes were chosen to give examples of microscope application, rather than do any specific biological claims.
Data exclusions	No data was excluded.
Replication	We do not claim any specific experimental findings, because our paper is focused on a new microscope design.
Randomization	Randomization is not relevant to our study for the reasons stated above.
Blinding	Randomization is not relevant to our study for the reasons stated above.

Reporting for specific materials, systems and methods

We require information from authors about some types of materials, experimental systems and methods used in many studies. Here, indicate whether each material, system or method listed is relevant to your study. If you are not sure if a list item applies to your research, read the appropriate section before selecting a response.

Materials & experimental systems

n/a	Involved in the study	
☐	☒	Antibodies
☒	☐	Eukaryotic cell lines
☒	☐	Palaeontology and archaeology
☐	☒	Animals and other organisms
☒	☐	Clinical data
☒	☐	Dual use research of concern
☒	☐	Plants

Methods

n/a	Involved in the study	
☒	☐	ChIP-seq
☒	☐	Flow cytometry
☒	☐	MRI-based neuroimaging

Antibodies

Antibodies used

Rabbit-anti-RFP antibody (Rockland, 600-401-379-RTU), donkey-anti-rabbit-Cy3 antibody (Jackson ImmunoResearch, 711-165-152), chicken-anti-GFP antibody (Aves Labs, GFP-1020), donkey-anti-chicken-AlexaFluor594 antibody (Jackson Immuno Research, 703-585-155), Cy3-conjugated monoclonal anti-α-smooth muscle actin antibody (Sigma Aldrich, C6198), Atto647N conjugated anti-GFP nanobooster (Chromotek, gba647n-100), antiAtp1a1 (DSHB, A5), anti-col2a1 (DSHB, II-II6B3) and anti-TNNT2 (DSHB, CT3), Alexa Fluor 555 goat anti-mouse IgG (405324, P4U/BioLegend UK), mouse anti-neurofilament (RMO270, Invitrogen 13-0700), goat anti-mouse IgG-Cy3 (Jackson ImmunoResearch 115-165-003).

Validation

Rabbit-anti-RFP antibody (Rockland, 600-401-379-RTU)
RTU Anti-RFP was prepared from monospecific antiserum by immunoaffinity chromatography using Red Fluorescent Protein (Discosoma) coupled to agarose beads followed by solid phase adsorption(s) to remove any unwanted reactivities. Expect reactivity against RFP and its variants: mCherry, tdTomato, mBanana, mOrange, mPlum, mOrange and mStrawberry. Assay by immunoelectrophoresis resulted in a single precipitin arc against anti-Rabbit Serum and purified and partially purified Red Fluorescent Protein (Discosoma). No reaction was observed against Human, Mouse or Rat serum proteins.
Validation references at https://www.rockland.com/categories/primary-antibodies/ready-to-use-rfp-antibody-pre-adsorbed-600-401-379-RTU/

Chicken-anti-GFP antibody (Aves Labs, GFP-1020)
Antibodies were analyzed by western blot analysis (1:5000 dilution) and immunohistochemistry (1:500 dilution) using transgenic mice expressing the GFP gene product. Western blots were performed using BlokHen® (Aves Labs) as the blocking reagent, and HRP-labeled goat anti-chicken antibodies (Aves Labs, Cat. #H-1004) as the detection reagent. Immunohistochemistry used tetramethyl rhodamine-labeled anti-chicken IgY.
Validation references at https://www.aveslabs.com/products/anti-green-fluorescent-protein-antibody-gfp

Cy3-conjugated monoclonal anti-α-smooth muscle actin antibody (Sigma Aldrich, C6198)
Monoclonal Anti-Actin, α-Smooth Muscle (mouse IgG2a isotype) is derived from the 1A4 hybridoma produced by the fusion of mouse myeloma cells and splenocytes of immunized BALB/c mice. The antibody (also known as anti-α-Sm-1) is specific for the single isoform of α-smooth muscle actin. It reacts specifically with α-smooth muscle actin in immunoblotting assays and labels smooth muscle cells in frozen or formalin-fixed, paraffin-embedded tissue sections.
Validation references at https://www.sigmaaldrich.com/CH/en/product/sigma/c6198

Atto647N conjugated anti-GFP nanobooster (Chromotek, gba647n-100)
The nanobody used in this study has been extensively validated. The manufacturer website provides more than 50 publications in which the nanobody has been used. In regards of the specific application in tissue clearing on rodents, shown in this manuscript, data and details of the protocol to use and validate the nanobody for this purpose is described here: PMID 36697871.
Specificity/Target: CFP, AcGFP, EGFP, GFP, GFP S65T, mClover, EGFP A206K, pHluorin, PA-GFP, sfGFP, TagGFP, TagGFP2, Citrine, Ecitrine, EYFP, Venus, YFP, Ypet.
Further validation references at https://www.ptglab.com/products/GFP-Booster-ATTO647N-gba647n.htm

Atp1a1 (DSHB, A5)
Antibody binds a cytosolic epitope on the alpha-subunit of all Na, K-ATPase isoforms. IP recognizes denatured ATPase only.
Confirmed Species Reactivity: Avian, Drosophila, Fish, Frog, Honeybee, Human, Insect, Mackerel, Mammal, Mosquito, Zebrafish
initial publication - PMID: 2540956
validated in Xenopus - PMID 34739029

col2a1 (DSHB, II-II6B3)
Epitope lies within the triple-helical domain of chick collagen type II.
Confirmed Species Reactivity: Avian, Bovine, Broad species, Chicken, Fish, Goat, Human, Mouse, Ovine, Quail, Rabbit, Rat, Shark, Xenopus, Zebrafish
initial publication - PMID: 7356475
validated in Xenopus - PMID 34739029

TNNT2 (DSHB, CT3)
CT3 recognizes the embryonic and adult cardiac isforms [PMID: 2358124]. CT3 cross-react with slow skeletal muscle TnT but doesn't recognize fast skeletal muscle TnT
Confirmed Species Reactivity: Broad species, Chicken, Fish, Human, Mouse, Rabbit, Rat, Xenopus
initial publication - PMID: 2358124
Validated in Xenopus - PMID:10644411

Mouse anti neurofilament NEFM Monoclonal Antibody (RMO-270)
Neurofilaments (NFs) are a type of intermediate filament (IF) expressed almost exclusively in neuronal cells, and in those cells most prominently in large axons. NFs, in most vertebrates, are composed of three different polypeptide chains with different molecular weights - neurofilament medium protein (NF-M), high (NF-H) and light protein (NF-L), which share sequence and structural similarity in a coiled-coil core domain, but differ in the length and sequence of their N-termini and more dramatically of their C-termini which in the case of NF-M and NF-H form the flexible extensions that link NFs to each other and to other elements in the cytoplasm. NF-M protein tail-mediated interactions of neurofilaments are critical for size and cytoskeletal architecture of axons, and are mediated, in part, by the highly phosphorylated tail domain of this protein. This antibody reacts with the 160 kD polypeptide subunit of human neurofilament. It specifically recognizes a phosphate-independent epitope in the tail (carboxy) domain of NF-M of most vertebrates and invertebrates.
Validation references at https://www.thermofisher.com/antibody/product/NEFM-Antibody-clone-RMO-270-Monoclonal/13-0700

Animals and other research organisms

Policy information about studies involving animals; ARRIVE guidelines recommended for reporting animal research, and Sex and Gender in Research

Laboratory animals	Mouse (age: P14, 2.8 months, 8 months, 10.8 months old), chicken (Day 9), Xenopus tropicalis frog (stage 58)
Wild animals	The study did not involve wild animals.
Reporting on sex	Sex-based analysis was not performed in this paper due to its focus on microscopy methods and application examples.
Field-collected samples	The study did not involve samples collected in the field.
Ethics oversight	Local regulatory authorities (Kantonales Veterinärsamt Zürich), local guidelines (TschV, Zurich) and the Swiss animal protection law (TschG). Ethical review board of the government of Upper Bavaria (Regierung von Oberbayern, Munich, Germany) and institutional guidelines of Klinikum der Universität München/Ludwig Maximilian University of Munich).

Note that full information on the approval of the study protocol must also be provided in the manuscript.
