# Supplementary material for: Benchtop mesoSPIM: a next-generation open-source light-sheet microscope for cleared samples
Source: Nat Commun. 2024 Mar 27;15:2679. doi: 10.1038/s41467-024-46770-2 (PMC10973490; doi:10.1038/s41467-024-46770-2)
Supplement: Supplementary file 3 — Description of Additional Supplementary Files [file 41467_2024_46770_MOESM3_ESM.pdf]

Description of Additional Supplementary Files

Supplementary Movie 1

Description: Contrast map of Olympus XLFluor 4x/0.28 objective based on imaging a 40lp/mm Ronchi grid through 20 mm of high-index medium (RI 1.52). The Z axis represents focus depth F [μm] scaled by 10x for demonstration purpose.

Supplementary Movie 2

Description: Mouse brain with spinal cord dissected from a 2.5-month-old Thy1-GFP line M animal, cleared with vDISCO and imaged at 5x magnification.

Supplementary Movie 3

Description: Whole mouse P14 stained with propidium iodide and cleared with vDISCO, imaged at 0.9x magnification.

Supplementary Movie 4

Description: Chicken embryo at E9 stage stained with M Anti-RMO270, G Anti-Mouse Cy3, cleared with BABB, imaged at 1.2x magnification.

Supplementary Movie 5

Description: Brain of APPPS-1 mouse, stained with hFTAA (amyloid plaques) and SMA-Cy3 (arterial vessels), cleared with iDISCO, imaged at 1.2x magnification.

Supplementary Movie 6

Description: Brain of Vglut2-Cre mouse with retrograde AAV cre-dependent variant of td-Tomato (ssAAV-retro/2-CAG-dlox-tdTomato (rev)-dlox-WPRE-bGHp(A), cleared with iDISCO, imaged at 4x magnification.

Supplementary Movie 7

Description: One hemisphere of Thy1-YFP AF-561 mouse, cleared with iDISCO, imaged at 5x magnification.

Supplementary Movie 8

Description: Piece of human cortex (occipital lobe V2) from 90 y.o. male donor (Occipital lobe 2), stained with neutral red and cleared with the MASH-NR protocol (Nissl staining with neutral red; ECi as immersion medium), imaged at 5x magnification.

Supplementary Movie 9

Description: Human cortex (part of occipital lobe V2) from 101 y.o. female donor (Occipital lobe 1), stained with methylene blue and cleared with the MASH-NR MB protocol (Nissl staining with methylene blue; ECi as immersion medium), imaged at 5x magnification.

Supplementary Movie 10

Description: Xenopus tadpole stage 58, stained with Atp1a1 AF-555, cleared with BABB, imaged at 5x.

Supplementary Movie 11

Description: Benchtop mesoSPIM assembly from modules in 50 minutes at EMBO Course “3D developmental imaging”, Oeiras, 2022. URL: https://youtu.be/4nsc5BLfjYc

Supplementary Movie 12

Description: Galvo assembly for Benchtop mesoSPIM excitation arm. URL: https://youtu.be/53Eq5iCVMGg
